# Unraveling the Role of MoO_x_ Clusters in Ternary Mo/Co_3_NiO_x_ for Boosting Acidic Oxygen‐Evolution Performance

**DOI:** 10.1002/advs.202511456

**Published:** 2025-12-10

**Authors:** Qiong Zeng, Jingjing Zhang, Sarvesh Manoj Jadhav, Yigui Wang, Zhiwen Li, Dequan Xiao, Gao Li

**Affiliations:** ^1^ University of Science and Technology of China Hefei 230026 China; ^2^ Dalian Institute of Chemical Physics Chinese Academy of Sciences Dalian 116023 China; ^3^ School of Chemistry and Chemical Engineering Inner Mongolia Normal University Hohhot 010018 China; ^4^ Center for Integrative Materials Discovery Department of Chemistry and Chemical and Biomedical Engineering University of New Haven West Haven CT 06525 USA

**Keywords:** acidic oxygen evolution reaction, Co_3_NiO_x_, DFT, MoO_x_ clusters, synergistic effect

## Abstract

Developing efficient transition metal electrocatalysts with low cost for the acidic oxygen evolution reaction (OER) confronts an enormous challenge. This work reveals the promising potential of ternary Mo/Co_3_NiO_x_ OER electrocatalysts, exhibiting good overpotentials of 249 mV@10 mA cm^−2^ and 429 mV@50 mA cm^−2^ and a small Tafel slope of 219 mV dec^−1^ in OER tests (in a 0.5 m H_2_SO_4_ electrolyte), much better than those for the corresponding com‐RuO_2_ (330 mV@10 mA cm^−2^ and 445 mV dec^−1^). Furthermore, it exhibits good durability, lasting for ≈200 hours at a current density of 10 mA cm^−2^, which can be attributed to the synergistic effect of the ternary heterostructure. The deactivation of Mo/Co_3_NiO_x_ catalyst is attributed to the loss of Ni species, which then leads to the structural destruction, corroborated by methods of in situ inductively coupled Plasma‐optical emission spectroscopy and X‐ray photoelectron spectroscopy. Finally, in situ attenuated total reflection‐surface enhanced infrared absorption spectroscopy (ATR‐SEIRAS) combined with DFT results show that the pristine Co_3_NiO_x_ prefers the adsorbate evolution mechanism (AEM), and proceeds via a synergistic interplay between AEM and lattice oxygen oxidation mechanism (LOM).

## Graphical abstract

The ternary Mo/Co_3_NiO_x_ outperforms commercial RuO_2_ in acidic OER tests and exhibits good durability (lasting for ≈200 hours@10 mA cm^−2^), which is attributed to alteration of AEM pathway (for Co_3_NiO_x_ carriers) to a synergistic interplay between AEM and LOM pathways.

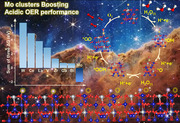

## 1 Introduction

The utilization of renewable energy for water electrolysis to produce hydrogen offers a reliable and sustainable pathway for generating clean hydrogen fuel, playing a significant role in the global energy transition.^[^ ^1^, ^2^, ^3^, ^4^ ^]^ However, the slow kinetics and insufficient stability of proton‐coupled electron transfer reactions in traditional electrolysis systems severely limit the overall efficiency of water splitting.^[^ ^5^, ^6^, ^7^ ^]^ Consequently, optimizing the performance of electrolysis systems is crucial, with the efficiency of the oxygen evolution reaction (OER) being particularly critical, as it serves as the rate‐determining step in the water splitting process.^[^ ^8^, ^9^, ^10^ ^]^ Water electrolysis systems featuring proton exchange membranes (PEM) have garnered widespread global attention due to their numerous advantages, such as high current density, high‐purity gas products, low ohmic losses, and excellent system compactness.^[^ ^11^, ^12^ ^]^ However, this technology necessitates the use of electrocatalysts that can operate stably in highly acidic environments and resist corrosion, presenting significant challenges for material design.^[^ ^13^, ^14^, ^15^ ^]^ Currently, iridium‐based catalysts, particularly IrO_x_, are regarded as the most practical electrocatalysts for OER in PEM systems due to their exceptional durability and efficiency.^[^ ^16^, ^17^ ^]^ Nevertheless, the scarcity and high cost of Ir (≈4240 USD/oz) limit its large‐scale application, driving researchers to explore more cost‐effective alternatives. Ruthenium (≈545 USD/oz) with one‐eighth price of Ir and positioned as a promising substitute has demonstrated commendable OER activity in acidic environments.^[^ ^18^, ^19^, ^20^ ^]^ However, the development of other low‐cost and efficient OER catalysts suitable for acidic environments remains a critical research objective.^[^ ^21^, ^22^, ^23^ ^]^

Recent progress has highlighted the urgent need for non‐noble metal catalysts that can operate efficiently and durably under acidic OER conditions, where stability remains a critical challenge. Various design strategies, including multi‐metal oxides, structural/electronic regulation, and metal‐organic‐framework (MOF)‐derived frameworks, have been proposed to balance catalytic activity and durability. Xu et al. provided a comprehensive review of such approaches, underscoring the importance of tailoring both composition and structure to address the dual requirements of activity and stability in acidic media.^[^ ^24^ ^]^ Meanwhile, several 3d transition metals, such as cobalt‐ (≈1 USD/oz) and nickel‐based catalysts, are considered promising alternatives to the currently used precious metal catalysts due to their low cost, high activity, and multivalent characteristics.^[^ ^25^, ^26^ ^]^ These properties could potentially overcome the performance limitations associated with large‐scale hydrogen production.^[^ ^27^, ^28^, ^29^ ^]^ Meanwhile, Sun et al. demonstrated that selecting customizable combinations of metal atoms as precursors from an extensive binuclear metal library enables the rational design of catalysts with tailored active sites.^[^ ^30^ ^]^ However, studies have shown that Co‐based catalysts undergo structural evolution and surface reconstruction under acidic OER conditions, which significantly affects their electrochemical behavior and catalytic activity.^[^ ^31^, ^32^, ^33^ ^]^ To enhance OER kinetics, refining the pre‐oxidation process of Co_3_O_4_ is an effective strategy.^[^ ^34^, ^35^ ^]^ For instance, Wang et al. introduced trace fluorine to precisely modulate the coordination environment of Co_3_O_4_ (Co_3_O_4_‐F_x_), which improves intrinsic activity and stability under acidic conditions.^[^ ^33^ ^]^ Ni‐based catalysts are less used in acidic OER due to their tendency to dissolve.^[^ ^36^, ^37^ ^]^ Xie et al. found that the incorporating Ni atoms in iridium sheets generates more oxygen atoms, thereby achieving better OER performance.^[^ ^38^ ^]^ Meanwhile under OER conditions, surface reconstruction produces the new active species, and targeted rational regulation of this reconstruction is key to constructing highly active catalysts.^[^ ^39^, ^40^ ^]^ Chen et al. discovered that the high‐valent Mo‐modulated PrIrMoO_x_ enabled directed and accelerated surface reconstruction to generate self‐terminating Ir‐O_bri_‐Mo active species, which exhibited high activity for acidic water oxidation.^[^ ^41^ ^]^ The doped Mo not only accelerates surface reconstruction but also enhances durability by buffering charge compensation, with the residual Mo inducing strong acidity in O_bri_, promoting the deprotonation of oxygen intermediates. The intrinsic OER activity of Co_3_O_4_ can be improved by modifying its redox properties.^[^ ^14^, ^42^, ^43^ ^]^ Therefore, investigating the relationship between redox properties and catalytic OER performance is particularly important for advancing the understanding of reaction mechanisms.

In our previous work, we successfully synthesized cobalt‐based clusters through ligand bonding, which exhibited remarkable electrochemical efficiency in the acidic OER.^[^ ^42^, ^44^, ^45^ ^]^ Herein, we prepared a homogeneous single‐phase Co_3_NiO_x_ solid solution, which is described as a nickel doped cobalt oxide with a key feature being its ability to retain the crystal structure. Subsequently, we introduced molybdenum clusters to modulate the electronic properties of Co_3_NiO_x_ carriers. The resulting Mo/Co_3_NiO_x_ demonstrated exceptional catalytic performance in the acidic OER, achieving overpotentials of 249 mV at 10 mA cm^−2^ and 429 mV at 50 mA cm^−2^. DFT simulations demonstrate that the catalytically active sites should be associated with the supported Mo species with higher oxidation states and the introduction of Mo mediates the rate limiting step of O* to O_2_* (for Mo/Co_3_NiO_x_) differing from that for bare Co_3_NiO_x_ carriers (the H‐removing step from *OH → O). These results underscore the potential of Mo/Co_3_NiO_x_ as a highly effective catalyst for electrocatalytic applications, particularly in acidic OER processes.

## 2 Results and Discussion

### 2.1 Synthesis and Characterizations of Mo/Co_3_NiO_x_

**Figure 1:**
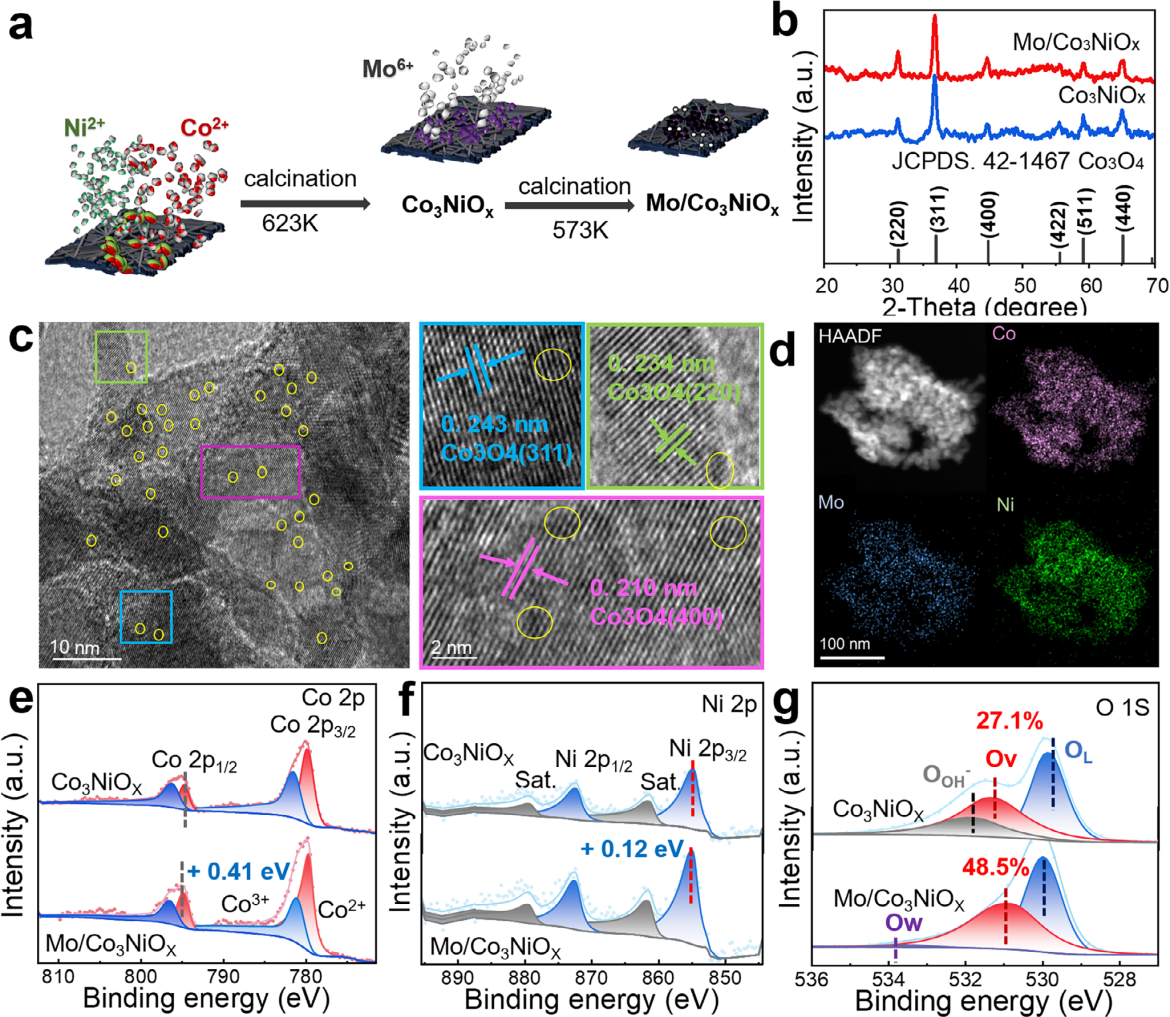
Structural and morphological characterization of Mo/Co_3_NiO_x_ electrocatalysts. a) Schematic illustration of the synthesis process of Mo/Co_3_NiO_x_ composites. b) XRD patterns. c) HRTEM image of Mo/Co_3_NiO_x_ catalyst; the magnified images are displayed alongside (MoO_x_ clusters highlighted by yellow circles). d) EDS elemental mapping (Co, pink; Mo, blue; Ni, green). High solution XPS spectra of Mo/Co_3_NiO_x_ and Co_3_NiO_x_: e) Co 2p, f) Ni 2p, and g) O 1s.

Based on the systematic characterization results in **Figure** **1**, we gain deep insights into the structural features and formation mechanism of Mo/Co_3_NiO_x_. The material synthesis involved two critical stages (Figure 1a): initial annealing of Co^2+^ and Ni^2+^ precursors at 623 K to form Co_3_NiO_x_, followed by an ammonium molybdate (VI) solution was drop‐coated onto the electrode precursor and subsequent annealing at 573 K to obtain Mo/Co_3_NiO_x_ (The molar ratio of Co:Ni:Mo was determined to be 3:1:0.8 by ICP analysis). X‐ray diffraction (XRD) analysis was performed to determine the crystalline structure of Mo/Co_3_NiO_x_ (Figure 1b). Six strong diffraction lines were observed at 2θ ≈ 19.0°, 31.3°, 38.5°, 44.8°, 59.3°, and 65.2°, which correspond to the planes (220), (311), (400), (422), (511) and (440) of cubic Co_3_O_4_ in the Fd‐3m (227) space group (JCPDS #42‐1467).^[^ ^42^ ^]^ Furthermore, Due to the highly dispersed nature of molybdenum ions as small particle clusters within the cobalt‐nickel oxide framework, no reflections corresponding to NiO_x_ species (e.g., NiO) was observed in the XRD patterns of these Mo/Co_3_NiO_x_ samples. This absence is attributed to the highly dispersed nature of Ni ions, which is likely present as small clusters or atomically distributed species within the cobalt–nickel oxide framework, rather than forming long‐range ordered NiO_x_ crystallites.^[^ ^46^, ^47^ ^]^ Subsequently, the Mo/Co_3_NiO_x_ were analyzed using transmission electron microscopy (TEM) to determine their elemental composition and morphology. TEM imaging revealed that most oxide particles ranged in size from 6 to 10 nm (Figure S1, Supporting Information). High‐resolution TEM (HR‐TEM) image reveals the presence of MoO_x_ clusters (highlighted by yellow circles) uniformly dispersed on the crystalline matrix (Figure 1c). These clusters exhibit nanoscale dimensions, with most diameters in the range of 1–2 nm (Figure S2, Supporting Information). These clusters are of nanometric size and appear to be anchored on or embedded within the host lattice without significant aggregation. The lattice fringes exhibit an interplanar distance of 0.243 nm, 0.234 nm, and 0.289 nm, which correspond to the (311), (220), and (400) planes of cubic Co_3_O_4_,^[^ ^48^ ^]^ respectively. Intriguingly, no lattice fringes for NiO_x_ species was observed, indicating that Ni atoms were successfully dispersed within the lattice of Co_3_O_4_.^[^ ^49^ ^]^ The small‐sized MoO_x_ clusters of 1‐2 nm are highly dispersed on the surface of Co_3_NiO_x_ carriers. Its intimate contact with Co_3_NiO_x_ carriers is expected to benefit catalytic performance by increasing the number of accessible active sites.^[^ ^50^ ^]^ Furthermore, the linear‐scanning profiles depicted in Figure S3 (Supporting Information) also confirmed that Mo clusters were successfully distributed within the lattice of Co_3_O_4_ (yellow circles indicate Mo clusters).^[^ ^51^ ^]^ The elemental mapping further confirms the simultaneous presence of Co, Ni, and Mo in the Mo/Co_3_NiO_x_ (Figure 1d).

**Figure 2:**
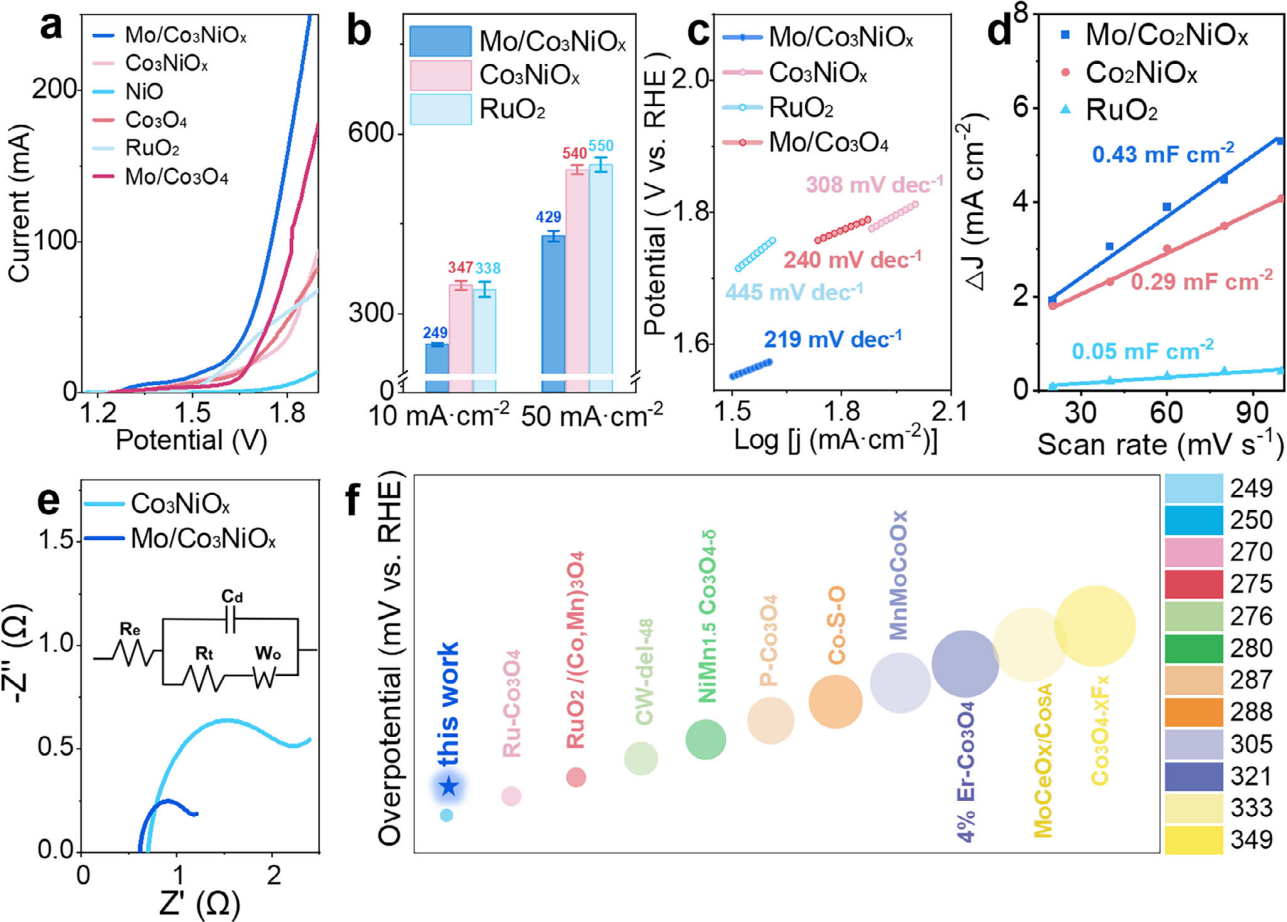
Catalytic OER performance. OER polarization curves of a) Mo/CoNiO_x_ composites, Co_3_NiO_x_, Co_3_O_4_, Mo/Co_3_O_4_, RuO_2_, and NiO reference samples in a 0.5 m H_2_SO_4_ (pH 0) solution. b) Overpotentials with deviation detections, c) Tafel plots, d) Cdl values, and e) Nyquist plots (at E_j=10_) of Mo/Co_3_NiO_x_ and Co_5_NiO_x_. f) Comparison of overpotentials at 10 mA cm^−2^ current density for different catalysts (Table S3, Supporting Information).

Next, X‐ray photoelectron spectroscopy (XPS) analysis was performed to investigate the chemical states and electronic structure of Mo/Co_3_NiO_x_ Figures S4 and S5 (Supporting Information). As shown in Figure 1e, the deconvolution of Co 2p3/2 signal revealed the existence of Co^3+^ (binding energy (BE): 781.1 eV) and Co^2+^ (Be: ≈779.6 eV) species in plain Co_3_NiO_x_. And a ≈0.4 eV shift was found in the Co 2p3/2 signals of Mo/Co_3_NiO_x_ (Co^3+^ (BE: ≈781.5 eV) and Co^2+^ species (779.9 eV)), indicating that more electrons accumulated around the Co upon Mo incorporation, resulting in higher Co valence states. The Co^3+^/Co^2+^ ratio was determined to be 0.49 for Mo/Co_3_NiO_x_ and 0.41 for Co_5_NiO_x_. The higher ratio of Co^3+^/Co^2+^ is attributed to the incorporation of high‐valence Mo, which can boost the OER performance.^[^ ^41^, ^48^, ^52^, ^53^ ^]^ Further, the high‐resolution Ni 2p spectrum of Mo/Co_3_NiO_x_ can be deconvoluted into four peaks: the peaks centered at 872.47 eV and 854.93 eV belong to Ni 2p_1/2_ and Ni 2p_3/2_ and another two broad peaks at 879.45 eV and 861.60 eV are identified as the typical satellite peaks (sat.) (**Figure** **2**f). Similarly, a blue shift by 0.12 eV was also observed in Ni 2p signals for Mo/Co_3_NiO_x_, compared with Co_3_NiO_x_ carrier, suggesting the creation of nickel vacancies led to charge redistribution that could lead to the increasing Ni^3+^ population^[^ ^38^, ^54^ ^]^ (Table S1, Supporting Information). For Mo 3d XP spectrum, and the B.E. peaks at ≈ 232.30 and 232.77 eV, and 235.4 and 236.0 eV are affiliated with Mo^5+^ and Mo^6+^ species, respectively (Figure S6, Supporting Information). The O 1s spectra can be divided into three peaks at 529.5, 530.40, and 532.92 eV, which belong to the lattice oxygen (O_L_), vacancy oxygen (O_v_), and adsorbed oxygen (O_a_), respectively (Figure 2g). The Ov proportion in Mo/Co_3_NiO_x_ (48.5%) is much higher than Co_3_NiO_x_ (27.1%), which should be attributed to the defects in the small‐sized MoO_x_ clusters. Therefore, the Mo incorporation leads to significant modulation of the electronic structure of Mo/Co_3_NiO_x_. The synergistic work of MoO_x_ clusters and Co_3_NiO_x_ carriers with more active sites may promote the OER activity in acidic media.^[^ ^41^, ^55^ ^]^

### 2.2 Electrochemical Performance Evaluation

To assess the electrochemical activity of Mo/Co_3_NiO_x_ samples toward electrocatalytic water splitting, acidic OER tests of the as‐synthesized composites deposited on CP were performed in a solution of 0.5 m H_2_SO_4_ (pH 0) employing a conventional three‐electrode setup. Before the measurements, the reference electrode was calibrated relative to the reversible hydrogen electrode (RHE)^[^ ^56^ ^]^ (Figure S7, Supporting Information). Due to the loading of Co and Ni elements, the linear sweep voltammetry (LSV) data were recorded after cyclic voltammetry (CV) activation to remove the oxidation peaks, thereby ensuring accuracy (Figure S8, Supporting Information). Mo/Co_3_NiO_x_ exhibited better catalytic efficiency compared to Co_3_O_4_, NiO, Co_3_NiO_x_, Mo/Co_3_O_4_, and RuO_2_^[^ ^15^ ^]^ (Figure 2a). Electrocatalytic measurements revealed that Mo/Co_3_NiO_x_ exhibited a superior OER activity, outperforming other Mo/CoNiO_x_ counterparts with varied Co:Ni molar ratios as well as those with a fixed Co:Ni ratio but different Mo loading (Figure S9, Supporting Information).

Specifically, the Mo/Co_3_NiO_x_ gave overpotentials of 249 and 429 mV at a current density of 10 and 50 mA cm^−2^, respectively (Figure 2b), outperforming these over RuO_2_ (338 mV@10 mA cm^−2^ and 550 mV@50 mA cm^−2^) and Co_3_NiO_x_ (347 mV@10 mA cm^−2^ and 540 mV@50 mA cm^−2^). Such superior OER activity of Mo/Co_3_NiO_x_ can be ascribed to the synergistic work of MoO_x_ clusters and Co_3_NiO_x_ carriers in Mo/Co_3_NiO_x_. Next, we correlated the Ov concentration with the corresponding OER activity. Specifically, Mo/Co_3_NiO_x_ with higher Ov proportion exhibits better activity, indicating that the Ov in Mo/Co_3_NiO_x_ plays a crucial role in promoting OER activity, vide infra. The superior OER activity over Mo/Co_3_NiO_x_ surface stems from its smallest Tafel slopes of 219 mV dec^−1^ (Figure 2c), which is considerably lower than these of Co_3_NiO_x_ (308 mV dec^−1^), Mo/Co_3_O_4_(240 mV dec^−1^), and RuO_2_ (445 mV dec^−1^); hence suggesting the favorable OER kinetics of Mo/Co_3_O_4_. Moreover, we examined the intrinsic activity of the catalyst by electrochemical active surface area (ECSA),^[^ ^57^ ^]^ which is linearly proportional to the double‐layer capacitance (Cdl) derived from CV curves with various sweep rates at the non‐faradic region (Figure S10, Supporting Information). As shown in Figure 2d, the Cdl value of Mo/Co_3_NiO_x_ was significantly larger than that of Co_3_NiO_X_, indicating an increase in active sites available for OER.

**Figure 3:**
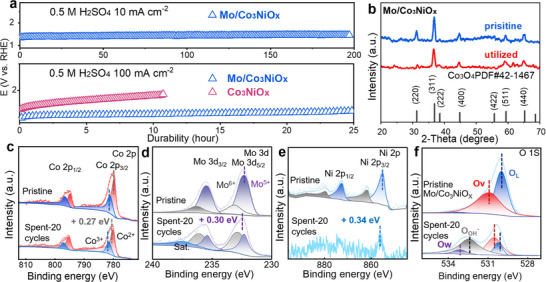
a) Potential required to reach 10 mA cm^−2^ in 0.5 m H_2_SO_4_ for Mo/Co_3_NiO_x_ measured using chronopotentiometry holds and the durability of Mo/Co_3_NiO_x_ at 100 mA cm^−2^ compared Co_3_NiO_x_. b) XRD patterns and XPS spectra of the pristine and utilized Mo/Co_3_NiO_x_ catalysts: c) Co 2p, d) Mo 3d, e) Ni 2p, and f) O 1s. Electrolysis conditions: in 0.5 m H_2_SO_4_ (pH 0) solution after 20 cycles at 100 mV s^−1^ at 298 K.

**Figure 4:**
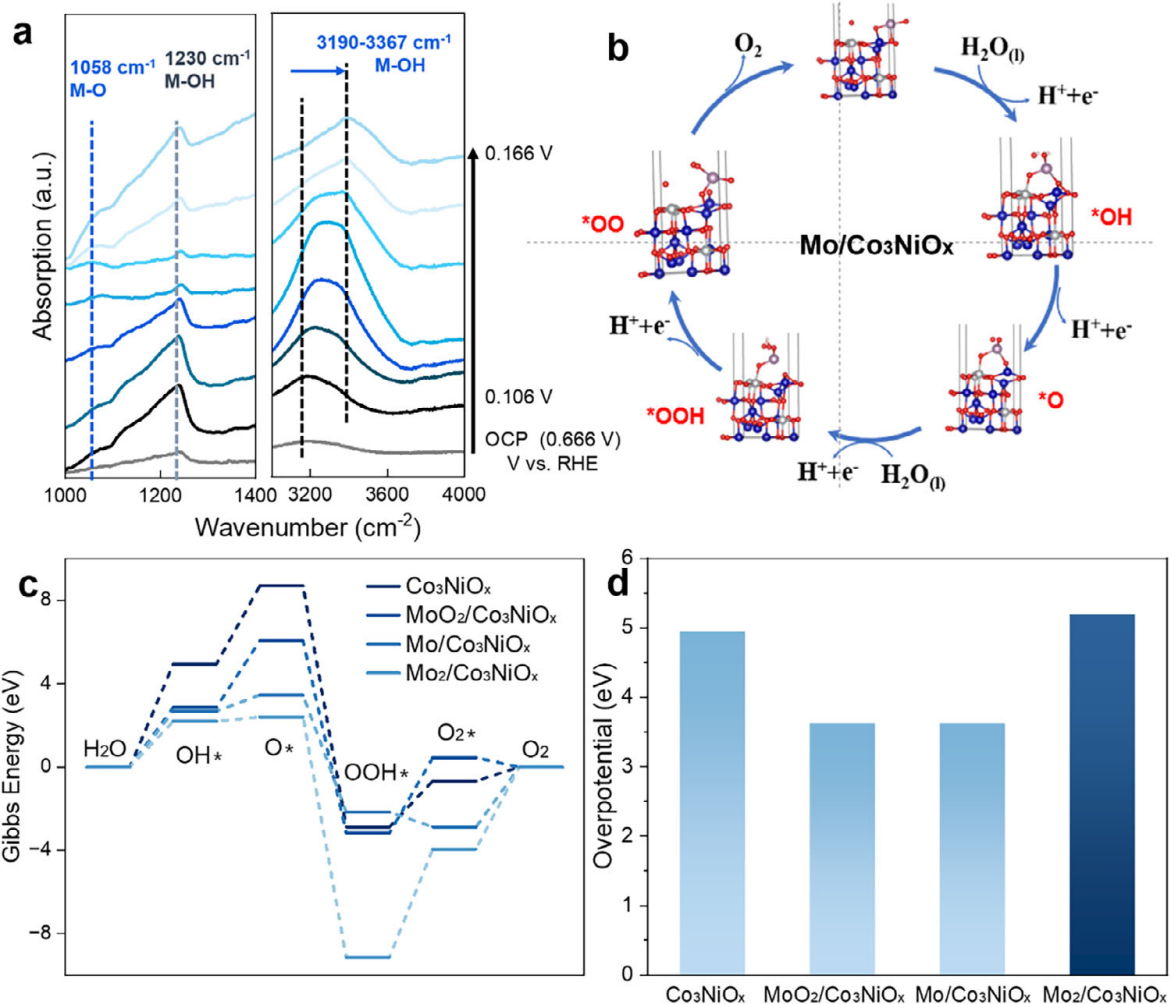
a) In situ ATR‐SEIRAS analysis of Mo/Co_3_NiO_x_. b) Five steps in the acidic oxygen evolution reaction (OER) with Mo/Co_3_NiO_x_ surface model (Co‐blue balls, Ni‐light gray balls, O‐red balls, Mo‐dark gray balls, and H‐small white ball). c) Different surface compositions: Co_3_NiO_x_, MoO_2_/Co_3_NiO_x_, Mo/Co_3_NiO_x_ and Mo_2_/Co_3_NiO_x_. d) The overpotential (eV) on four different surfaces.

In order to enhance our understanding of the electrode kinetics during OER, electrochemical impedance spectroscopy (EIS) was investigated at open circuit voltage E_j = 10_ with frequencies ranging from 10^6^ Hz to 10 mHz (**Figure** **3**e; Table S2, Supporting Information). The charge‐transfer resistances (R_ct_) of Mo/Co_3_NiO_x_ and Co_3_NiO_x_ was determined to be 0.49 Ω, and 1.35 Ω at E_j = 10_ (j stands for current density of 10 mA cm^−2^), respectively. The smaller R_ct_ means better electron‐transfer kinetics during OER.^[^ ^45^ ^]^ Figure 2f shows the comparison of current advanced OER catalysts in terms of their activity (Table S3, Supporting Information), substantiating that our Mo/Co_3_NiO_x_ electrocatalysts exhibited promising electrochemical performance in acidic OER. Next, the durability of Mo/Co_3_NiO_x_ catalyst to withstand acidic electrolytes is evaluated by chronopotentiometry (CP) measurements at the constant current densities of 10 and 100 mA cm^−2^ in 0.5 m H_2_SO_4_ (pH 0) (**Figure** **4**a). The Mo/Co_3_NiO_x_ catalyst exhibited the potential remaining at ≈1.46 V@10 mA cm^−2^ (for over 200 hours) and 1.72 V@100 mA cm^−2^ (25 hours), substantially outperforming the plain Co_3_NiO_x_ carriers, which should be attributed to the anchored MoO_x_ clusters. These experimental outcomes substantiate that Mo/Co_3_NiO_x_ electrocatalysts exhibited promising electrochemical activity and superior durability for OER.

### 2.3 Study of Catalyst Deactivation

Furthermore, we investigated the activity loss of Mo/Co_3_NiO_x_ electrocatalysts during the electrochemical process by comparing the pristine and utilized catalysts. All the intensity of XRD patterns of the utilized Mo/Co_3_NiO_x_ catalyst became weaker (Figure 3b), indicating the deformation of Mo/Co_3_NiO_x_ structure that significantly causes the deactivation of OER activity. The XPS results showed that the Co^3+^ and Mo^5+^ species were converted to Co^2+^ and Mo^6+^ species during the OER process (Figure 3c,d; Table S4, Supporting Information).^[^ ^58^, ^59^ ^]^ It has been demonstrated that introducing cation deficiency effectively promotes lattice‐oxygen participation by enhancing oxygen mobility and altering the bulk redox environment, thereby accelerating OER kinetics.^[^ ^60^ ^]^ And the Co 2p and Mo 3d of utilized Mo/Co_3_NiO_x_ shifted toward higher binding energy of ≈ 0.27 and 0.30 eV, demonstrating that more electrons are accumulated around Co atoms due to the oxidation behavior of Co^2+^ and Mo^5+^ species bonding with OH* (LOM mechanism) during the OER.^[^ ^10^, ^32^ ^]^ Interestingly, the surface hydroxyl species (BE: 532.38 eV) appeared and was largely enhanced (up to 44%, Table S4, Supporting Information) in the utilized Mo/Co_3_NiO_x_ catalyst (Figure 3e), signifying the surface amorphous structure of the utilized oxide electrocatalyst, which is well consistent with the observations in XRD results. More importantly, the Ni species are almost detached from the Mo/Co_3_NiO_x_ during the acidic OER tests, corroborated by the silent signals in the Ni 2p XPS spectrum of the utilized Mo/Co_3_NiO_x_. This could be ascribed to the compensatory effect of Ni species in preserving the structural stability of the catalyst lattice (Figure 3e; Figure S11, Supporting Information). Furthermore, we applied ICP‐OES method to monitor the Ni concentrations in the electrolyte during the durability tests: the Ni concentrations increased from ≈2.215 mg L^−1^ (1 hour) to 2.954 mg L^−1^ ppm (5 hours) and to 4.25 mg L^−1^ (25 hours). Thus, these results clearly manifested that the loss of Ni species in Mo/Co_3_NiO_x_ resulted in its structural destruction, which causes the deactivation of the catalyst in OER test (Figure S12 and Tables S5 and S6, Supporting Information). We also propose that the initial leaching of Mo arises from the dissolution of surface‐exposed Mo species under acidic conditions, while the Mo species coordinated within the Co–Ni framework remain intact and continue to contribute to the synergistic effect with Co and Ni.

### 2.4 Mechanism Study

To acquire the information of oxygen intermediates for a more comprehensive mechanistic understanding, the in situ attenuated total reflection‐surface enhanced infrared absorption spectroscopy (ATR‐SEIRAS) was performed using a home‐made electrochemical cell. The distinct vibrational signatures at 1230 and 3200–3500 cm^−1^ are assigned to be O‐O stretching of OOH and O‐H stretching of M‐OH, respectively (Figure 4a). The potential‐dependent shift of the O‐H stretching band (from 3200 to 3400 cm^−1^) suggests the deprotonation of surface hydroxyl groups, a critical step in the OER mechanism. Direct experimental evidence has confirmed the participation of lattice oxygen in the OER pathway, distinguishing LOM from the conventional adsorbate evolution mechanism (AEM) and underscoring the role of lattice engineering in enhancing activity.^[^ ^61^ ^]^ We also performed ATR‐SEIRAS experiments on the Co_3_NiO_x_ without Mo loading. The spectral features at 1235 and 3158–3239 cm^−1^ were assigned to the O–H stretching vibrations of M–OH species, indicating the exclusive involvement of the AEM mechanism (Figure S13, Supporting Information). The combined characterization results demonstrate that the introduction of Mo atoms generates additional surface defects on the catalyst, thereby promoting the participation of lattice oxygen in the reaction. Thus, the in situ spectroscopic and electrochemical results clearly demonstrate that the acidic OER over Mo/Co_3_NiO_x_ electrocatalysts should proceed via a synergistic interplay between the lattice oxygen oxidation mechanism (LOM) and adsorbate evolution mechanism (AEM).

Finally, to elucidate the acidic mechanism, DFT was applied to explore the corresponding geometric and electronic structure of Mo/Co_3_NiO_x_. The acidic OER involves four one‐electron transfer steps (Figure 4b). We first compared the reaction free energies for reaction steps on the Co_3_NiO_x_ (100) surface (Figure 4c). The step (H_2_O to OH*) or the step (OOH* to O_2_*) is the rate‐limiting step. Both Mo and MoO_2_ on Co_3_NiO_x_ could reduce the overpotential of the Co_3_NiO_x_ itself (Figure 4d). The Mo_2_/ Co_3_NiO_x_ catalyst slightly increases the overpotential (from 4.95 to 5.19 eV, Table S7, Supporting Information), possibly due to the lower number of O‐atoms around Mo (Figure S12, Supporting Information). We also used binding energies for OH*, O*, and OOH* to estimate the overpotentials. The loading of Mo, Mo_2_, or MoO_2_ on top of Co_3_NiO_x_ all reduces the overpotentials (from 2.70 to lower values) of acidic OER reaction (Table S8, Supporting Information). We then loaded MoO, MoO_2_, MoO_3_, and MoO_4_ on top of Co_3_NiO_x_ and used two Ni sites on the surface to accommodate intermediates OH*, O*, OOH*, OO*, and H_2_O*. It turns out that the intermediates stay on two Ni sites with MoO_4_ and MoO_3_, but further complexes with Mo site for MoO and MoO_2_ loaded catalysts (Figure S14, Supporting Information). In the meantime, the overpotentials are reduced significantly when intermediates complex with Mo vacancies, especially for MoO loaded catalysts, in which the overpotential reduces from 4.67 to 2.69 V using the one‐electron transfer model and from 2.13 V on Co_3_NiO_x_ to 0.39 V using binding energy method (Table S8, Supporting Information).

Without using the vacancies on MoO_x_ particles, as in the case of MoO_4_/Co_3_NiO_x,_ a small but consistent reduction in overpotential was confirmed in possible binding positions. MoO_4_/Co_3_NiO_x_ shifts d‐pDOS of Co and Ni to lower energy (Figure S15, Supporting Information) in comparison with Co_3_NiO_x_, and the Mo atoms gain more electron density by accepting electrons from coordinated O atoms through their d‐orbitals (Table S9, Supporting Information),^[^ ^62^ ^]^ indicating higher oxidation states of Mo help remove H from OOH* or OH*, and significantly reduces the over potential in the acidic OER reactions.

We also did calculations for a series of metals (Mo, Ce, La, Zr, Bi, Ga, V, and W) on Co_5_NiO_x_ (OER polarization curves of M/Co_5_NiO_x_ were exhibited in Figure S16, Supporting Information). In comparison with Co_5_NiO_x,_ all M/Co_5_NiO_x_ (except for M = V, W) have reduced overpotentials (Table S10, Supporting Information). It was found that the step to produce OH* is rate‐limiting step (4.15 eV) for Mo/Co_5_NiO_x_. The Mo/Co_5_NiO_x_ is outstanding among the M/Co_5_NiO_x_ catalysts, possibly because of MoO_n_’s ability to form a complex with OH through vacancy and help remove H.

## 3 Conclusion

In summary, we have prepared a promising Mo/Co_3_NiO_x_ electrocatalyst for the sustainable acidic oxygen evolution reaction. XRD combined with TEM analysis well confirms the structural composition of MoO_x_ clusters of 1–2 nm and Co_3_NiO_x_ solid solution carriers of ≈7 nm. Mo/Co_3_NiO_x_ showed excellent activity (249 mV@10 mA cm^−2^), outperforming Co_3_O_4_, Co_3_NiO_x_, and RuO_2_ references in the OER electrocatalysis. Moreover, the anchored MoO_x_ clusters significantly enhance the catalyst's stability during acidic OER. Followingly, the XPS analysis showed that the high‐valence Co^3+^ and Mo^6+^ and oxygen vacancies in the Mo/Co_3_NiO_x_ play an essential role in OER. Furthermore, in situ spectroscopic and corroborative DFT results validated that Mo/Co_3_NiO_x_ electrocatalysts employ a synergistic interplay between the lattice oxygen oxidation mechanism and adsorbate evolution mechanism during the acidic OER. The anchored Mo species should be the catalytically active sites: the higher oxidation states of Mo species, the better catalytic performance. In all, this study offers valuable guidance for designing efficient OER catalysts, highlighting Mo/Co_3_NiO_x_ as a promising candidate.

## Conflict of Interest

The authors declare no conflict of interest.

## Supporting information



Supporting Information

## Data Availability

The data that support the findings of this study are available from the corresponding author upon reasonable request.
