# Supplementary material for: Unraveling the Role of MoOx Clusters in Ternary Mo/Co3NiOx for Boosting Acidic Oxygen‐Evolution Performance
Source: Adv Sci (Weinh). 2025 Dec 10;13(5):e11456. doi: 10.1002/advs.202511456 (PMC12850222; doi:10.1002/advs.202511456)
Supplement: Supplementary file 1 — Supporting Information [file ADVS-13-e11456-s001.docx]

**Supporting Information**

**Unraveling the role of MoO_x_ clusters in** **ternary Mo/Co_3_NiO_x_ for boosting** **acidic oxygen-evolution performance**

Qiong Zeng,^1,3#^ Jingjing Zhang,^2,3#^ Sarvesh Manoj Jadhav,^4#^ Yigui Wang,^4^ Zhiwen Li,^2,3^ Dequan Xiao,^4*^ Gao Li^2,3*^

1. University of Science and Technology of China, Hefei 230026, China

2. School of Chemistry and Chemical Engineering, Inner Mongolia Normal University, Hohhot 010018, China

3. Dalian Institute of Chemical Physics, Chinese Academy of Sciences, Dalian 116023, China

4. Center for Integrative Materials Discovery, Department of Chemistry and Chemical and Biomedical Engineering, University of New Haven, West Haven, CT 06525, USA

^#^Q.Z., J.Z., and S.M.J. contribute equally to this work.

^*^Email: [DXiao@newhaven.edu](mailto:DXiao@newhaven.edu) (D.X.); li_gao82@yeah.net (G.L.)

Experimental

Electrocatalyst preparation. As shown in Fig. 1a, a 2 × 4 cm^2^ CP substrate were ultrasonically cleaned in ethanol, acetone, and ultrapure water, followed by treatment with a 10% oxalic acid solution for 4 hours to enhance their hydrophilicity. The Co_3_NiO_x_ support was synthesized in ultrapure water using cobalt (II) nitrate and nickel (II) acetate as catalysts. The resulting solution was drop-casted onto the substrate and, subsequently dried in an oven at 180°C for 10 minutes. After rinsing, the substrate was subjected to sonication for 10 seconds to effectively remove any loosely attached particles. The drop-casting, drying, and cleaning steps were repeated to ensure complete and consistent coverage. The electrode precursor was subsequently calcined at 623 K for 2 hours in a tubular furnace. Following this, an ammonium molybdate (Ⅵ) solution was drop-coated onto the electrode precursor, which was then subjected to further calcination at 573 K for 2 hours. This process resulted in a catalyst loading of approximately 0.05 mg on the CP substrate, with a geometric area of 0.25 cm^2^.

Characterization. The powder X-ray diffraction (XRD) patterns were conducted on an X-ray powder diffractometer (SmartLab, Rigaku Corporation, Tokyo, Japan) with Cu Kα radiation at 40 kV and 30^o^ mA from 10^o^ to 70^o^ at a scan rate of 10^o^ min^-1^ to identify the phase structure of these electrodes. X-ray photoelectron spectroscopy (XPS) analyses were conducted on a Theta Probe system (base pressure: ~ 10^−8^ Pa), equipped with a Phoibos 100 hemispherical analyzer and XR 50 X-ray source (SPECS GmbH) operated in the constant pass energy mode at 50 eV. Using Casa XPS, all spectra were corrected by the C 1s signal (284.8 eV). Subsequently, peaks were fitted after a Shirley-type background subtraction utilizing a mixed Gauss-Lorentz method, with peak positions and full-width half maxima (FWHM) left unconstrained. The concentration of the surface Co^3+^ (Co^3+^% = A_Co3+_/(A_Co3+_ + A_Co2+_)), Ni^2+^ (Ni^2+^% = A_Ni_^2+^/(A_Ni_^2+^ + A_Ni_^3+^)), Mo^5+^ (Mo^5+^% = A_Mo_^5+^/( A_Mo_^5+^ + A_Mo_^6+^)) and surface oxygen vacancies (O_v%_ = A_Ov_/(A_Ov_ + A_Ol_ + A_Oa_)) was calculated based on the area under the peaks of Co 2p, Mn 2p, Mo 3d and O 1s, respectively. Note that A*_i_* represents the peak area of the *i* species. Inductively Coupled Plasma-Optical Emission Spectroscopy (ICP-OES) was carried out on ICP-8100 (Shimadzu, Japan). The sample of Mo/Co_y_NiO_x_ were dissolved in an aqua regia solution. The actual ratios of Co/Ni/Mo in the electrocatalyst were determined to be 3/1/0.6 (for Mo/Co_3_NiO_x_ by ICP-OES). Transmission electron microscopy (TEM) and high-angle annular dark-field scanning transmission electron microscopy (HAADF-STEM) were carried out on Thermo Fisher FEI Talos F200x (Thermo F -0wq.0*entific, USA). Transmission XAS measurements were performed on a laboratory device (easyXAFS300, easyXAFS LLC), which is based on Rowland circle geometries with spherically bent crystal analyzers (SBCA) and a silicon drift detector. Si (5,5,1) SBCA was used for Mn measurement at 35 kV, and Si (5,3,3) SBCA was used for Co measurement at 30 kV. The powder samples were thoroughly ground and mixed with polyethylene glycol (PEG) using an agate mortar and pestle and pressed into Ø = 13 mm pellets. The pressed pellets were then sandwiched by Kapton tapes.

Electrochemical measurements. All the electrochemical measurements were carried out using an electrochemical workstation CHI 760E by a standard three-electrode system with a platinum electrode (Tianjin Aida Hengsheng Technology Development Co., Ltd.) as the counter electrode and Hg/Hg_2_Cl_2_ electrode (saturated KCl solution) as the reference electrode. The potential of the Hg/Hg_2_Cl_2_ reference electrode was corrected using a reversible hydrogen electrode (RHE) after the experiments. The working electrode was firstly cycled for 20 segments at 100 mV s^−1^ in 0.5 M H_2_SO_4_ (pH 0) to activate the electrode. And then the linear sweep voltammetry (LSV) was recorded at a scan rate of 5 mV s^−1^. The current density was calculated based on the geometric area (0.25 cm^2^) of the electrode exposed to the electrolyte solutions. All the potentials were referred to reversible hydrogen electrode by calibrating the reference electrodes using Pt sheets (purchased from Aldrich) as both the working and counter electrode in corresponding solutions. The iR compensation was performed manually after the measurement for 80% of the resistance (R).

In-situ ATR-SEIRAS tests. The in-situ attenuated total reflection surface-enhanced absorption spectroscopy (ATR-SEIRAS) measurements were carried out on Bruker 70 V Fourier-transform infrared (FTIR) spectrometer. The measurement featured a spectral resolution of 8 cm−1, with 64 interferograms co-added for each spectrum. The preparation of working electrodes comprised of two steps: firstly, an ultra-thin Au film was chemical deposited on the Si crystal to enhance the IR signal and facilitate electron conduction; secondly, the catalyst CP was onto the Au film. The Si crystal loaded with catalyst CP was placed onto a spectro-electrochemical three-electrode cell. Ag/AgCl electrode and Pt wire served as the reference and counter electrodes, respectively. The 0.5 M H2SO4 solution was used as the electrolyte. All SEIRAS spectra were obtained during the LSV test.

DFT calculations. The periodic density functional theory (DFT) was used for geometry relaxation and energy calculations with the Vienna Ab-initio Simulation Package (VASP). The projector augmented wave (PAW) method was used to compute the interactions between ion cores and valence electrons. The generalized gradient approximation (GGA+U) with the Perdew-Burke-Ernzerhof (PBE) functional were for the exchange correlation interactions, the strong on-site d-Coulomb interaction U (in eV) added to d-electrons were 3.32 (Co), 0.0 (Ce), 0.0 (Ga), 0.0 (La), 4.38 (Mo), 6.3 (Ni), 3.25 (V), 6.2 (W), and 0.0 (Zr), respectively. The DFT-D3 dispersion correction was used for energy-related calculations. A kinetic cutoff of 520 eV was used for the plane wave basis set. The geometry relaxations were conducted by a conjugate-gradient method with the convergence of 0.0014 eV/atom in energy and 0.02 eV/Å in force. A 4 × 4 × 3 gamma grid k-point mesh was used to sample the Brillouin zone. The optimized (100) surface of Co_3_O_4_ was adopted as the model for simulating reaction pathways. The unit cell of Co_3_O_4_ (100) surface contains 12 Co atoms and 16 O atoms. The Ni-doped Co_3_O_4_ was prepared by replacing several Co atoms in the unit cell. Mo atoms were placed on top of the (100) surface. During the optimization, the upper-layer atoms were allowed to relax and the atoms in the bottom layers were fixed to match the bulk structure. A vacuum layer of 20 Å was used along the c direction normal to the surface to avoid periodic interactions. The Gibbs free energy was calculated based on the four-electron OER mechanism proposed by Nørskov ^[1]^, where the corresponding formulas are listed the followings:

| H_2_O + * → HO* + (H* + e^−^) | ∆G(OH*) = ∆G(OH) − ∆G (H_2_O) – eU |
| --- | --- |
| OH* → O* + (H* + e^−^) | ∆G(O*) = ∆G(O) − ∆G(OH) – eU |
| H_2_O + O* → OOH* + (H* + e^−^) | ∆G(OOH*) = ∆G(OOH) − ∆G(O) − eU |
| OOH* → O_2_ + * + (H* + e^−^) | ∆G(O_2_*) = ∆G(O_2_) − ∆G(OOH) − eU |

The applied overpotentials were set as 0 and 1.23 V vs. reversible hydrogen electrode (RHE), respectively. The frequencies of adsorbates were calculated to obtain zero-point energy (ZPE) and entropy (S), so the thermal correction to G(T) could be evaluated by ZPE - T*S or by ZPE + ΔU(0→T) – TS [58]. The discussions in text were based on the latter. The overpotentials were taken as max[∆G(OH*), ∆G(O*), ∆G(OOH*), ∆G(O_2_*)]/e.

Another way to estimate overpotentials is through the binding energies of OH*, OOH*, and O*[45]. The binding Gibbs energies are calculated as follows:

| ΔG^0^_OH*_ = G^0^_OH*_ - G^0^(*) - (G^0^_H2O_-1/2G^0^_H2_) |
| --- |
| ΔG^0^_HOO*_ = G^0^_OOH*_ - G^0^(*) - (2G^0^_H2O_-3/2G^0^_H2_) |
| ΔG^0^_O*_ = G^0^_O*_ - G^0^(*) - (G^0^_H2O_-G^0^_H2_) |

The theoretical overpotential at standard conditions is given by

$ղ^{\boldsymbol{OER}}\boldsymbol{=}\left\{ \frac{\max\left[ \left( \boldsymbol{\Delta}\boldsymbol{G}_{\boldsymbol{O*}}^{\boldsymbol{0}}\boldsymbol{-\Delta}\boldsymbol{G}_{\boldsymbol{OH*}}^{\boldsymbol{0}} \right)\boldsymbol{, 3.2 eV-}\left( \boldsymbol{\Delta}\boldsymbol{G}_{\boldsymbol{O*}}^{\boldsymbol{0}}\boldsymbol{-\Delta}\boldsymbol{G}_{\boldsymbol{OH*}}^{\boldsymbol{0}} \right) \right]}{\boldsymbol{e}} \right\}\boldsymbol{-1.23 V}$

Statistical Analysis

All experimental data were collected from at least three independent measurements unless otherwise stated.

1. Data preprocessing. Raw electrochemical and spectroscopic data were checked for consistency and reproducibility. Outliers beyond ±2 SD of the mean were excluded. No transformation or normalization beyond baseline correction was applied.
2. Data presentation. Results are presented as mean ± standard deviation (SD). Error bars in the figures represent the SD of replicate measurements.
3. Sample size (n). For each electrochemical measurement (e.g., LSV, Tafel, EIS), a minimum of n = 3 independent electrodes were tested. For ICP-OES quantification, n = 2 parallel samples were measured per time point. For XPS and in-situ IR, at least n = 2 repeated scans were performed to confirm reproducibility.
4. Statistical methods. Differences between catalyst groups (e.g., different Mo loading or Co:Ni ratios) were assessed using one-way ANOVA followed by a Tukey’s post-hoc test for multiple comparisons. Where only two groups were compared, an unpaired two-tailed Student’s t-test was applied. Statistical significance was set at α = 0.05. Significance levels in the figures are indicated as P < 0.03
5. Software. Statistical analyses and graphing were performed using OriginPro 2025b (OriginLab) and powerpoint.

**
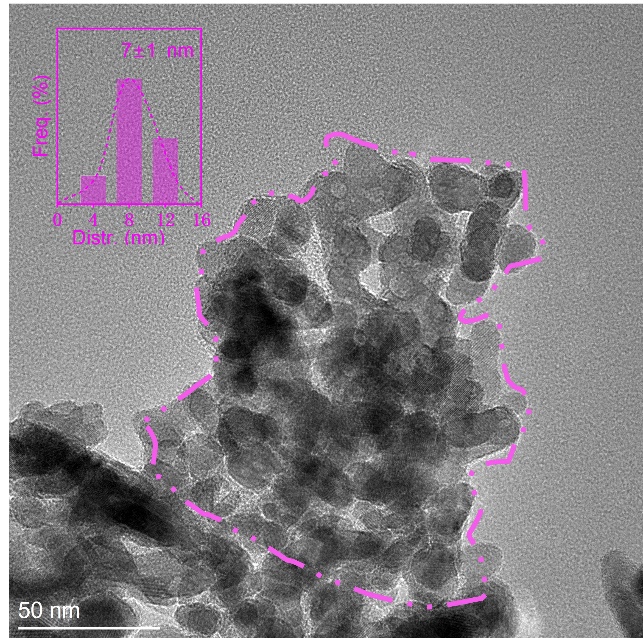
**

**Figure S1.** TEM image of the synthesized nanoparticles, showing uniformly distributed nanocrystals with an average particle size of 7±1 nm. The inset displays the corresponding particle size distribution histogram derived from statistical analysis.

**
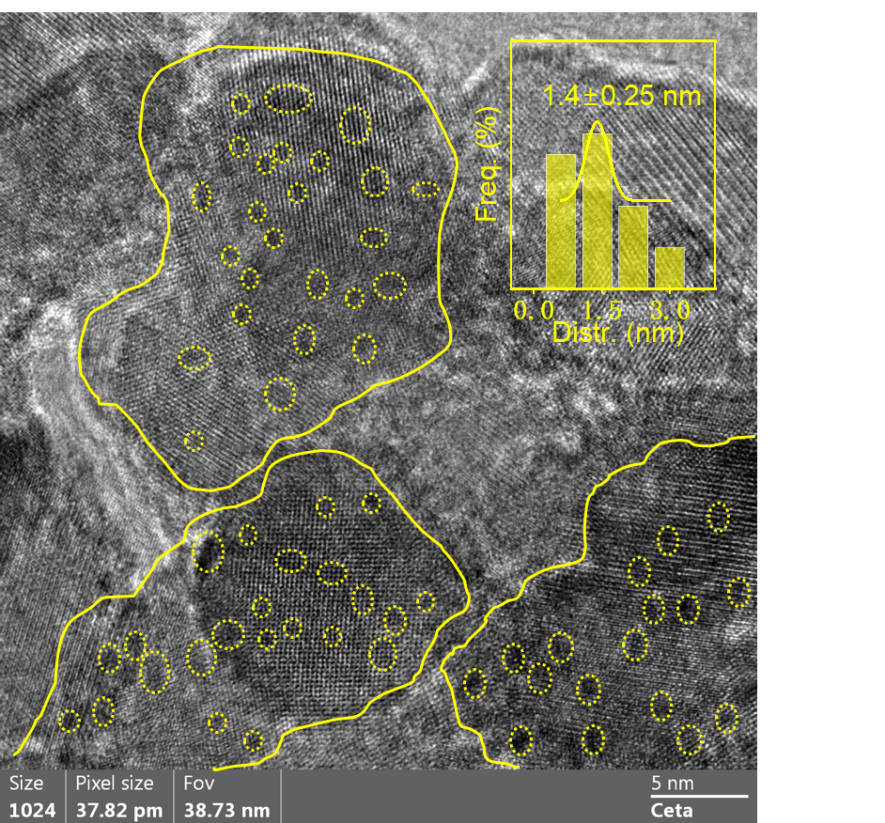
**

**Figure S2.** High-resolution transmission electron microscopy (HRTEM) image showing uniformly dispersed MoO_x_ nanoparticles (highlighted with yellow dashed circles) on the catalyst surface. The average particle size is ~1.4 ± 0.25 nm. The yellow solid lines outline regions enriched with nanoparticles. The inset displays the particle size distribution histogram, indicating that most particles fall within the 1–2 nm range.


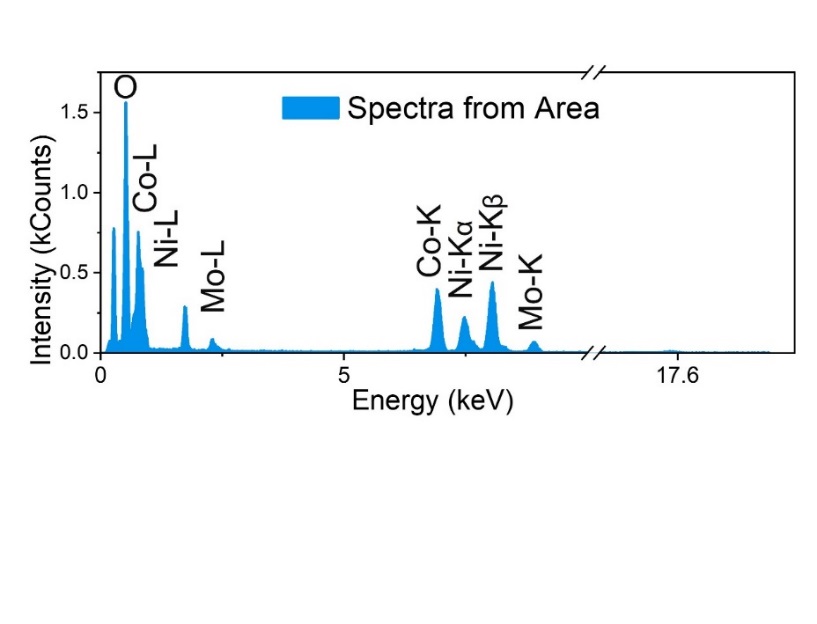


**Figure S3.** EDS spectrum collected from the selected area, showing characteristic peaks corresponding to O, Co, Ni, and Mo elements. The presence of both L-shell and K-shell emission lines indicates the coexistence of multiple elements in the analyzed region.

**
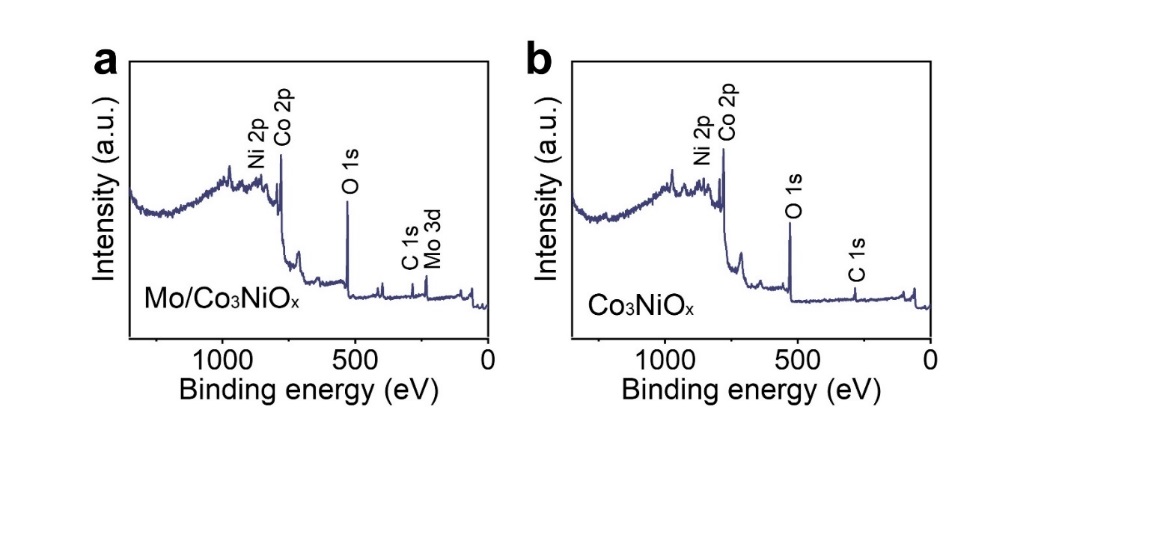
**

**Figure S4.** The full survey spectra of Co_3_NiO_x_ (a) and Mo/Co_3_NiO_x_ (b).


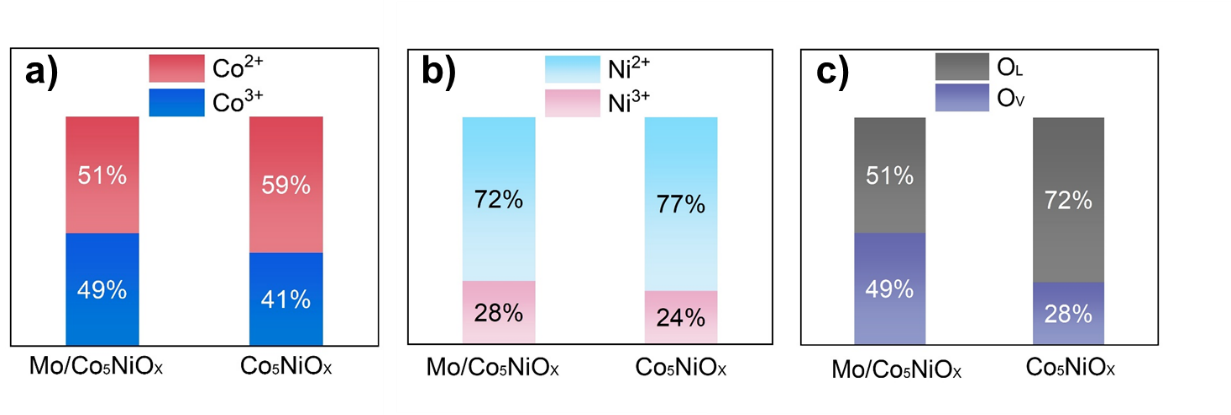


**Figure S5.** The ratios of (a) Co^2+^:Co^3+^, (b) Ni^2+^/Ni^3+^ and (c) O_L_:O_V_ in Mo/Co_3_NiO_x_ and Co_3_NiO_x_, analyzed by high-resolution XPS spectra (Fig. 1e-g).

**
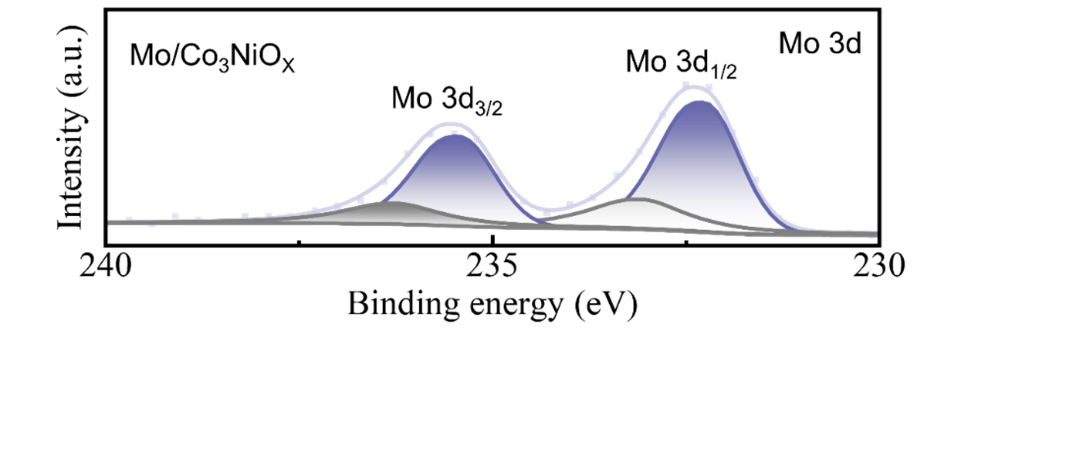
**

**Figure S6.** Mo 3d XPS spectrum of the pristine Mo/Co_3_NiO_x_.

**
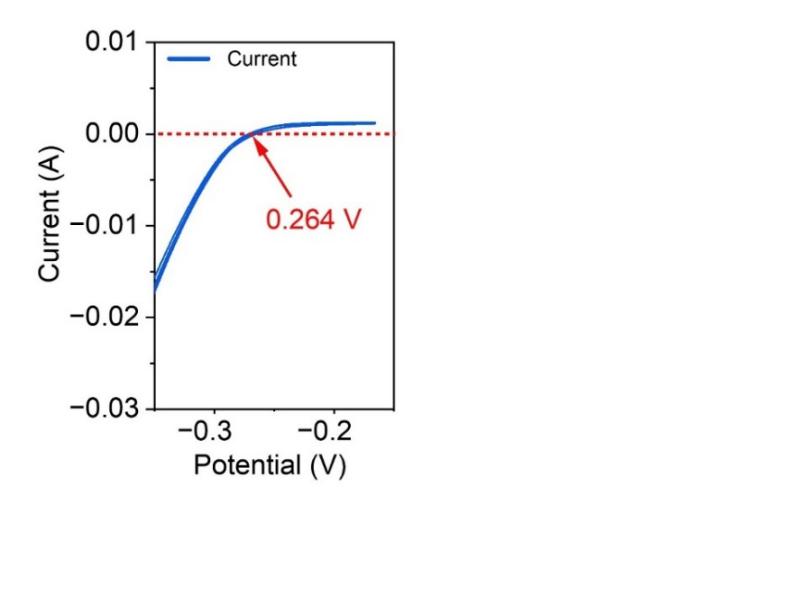
**

**Figure S7.** The calibration CV curve with the scan rate of 1 mV/s for the reference electrode (Hg/Hg_2_Cl_2_) in a 0.5 M H_2_SO_4_ (pH 0) solution at room temperature.


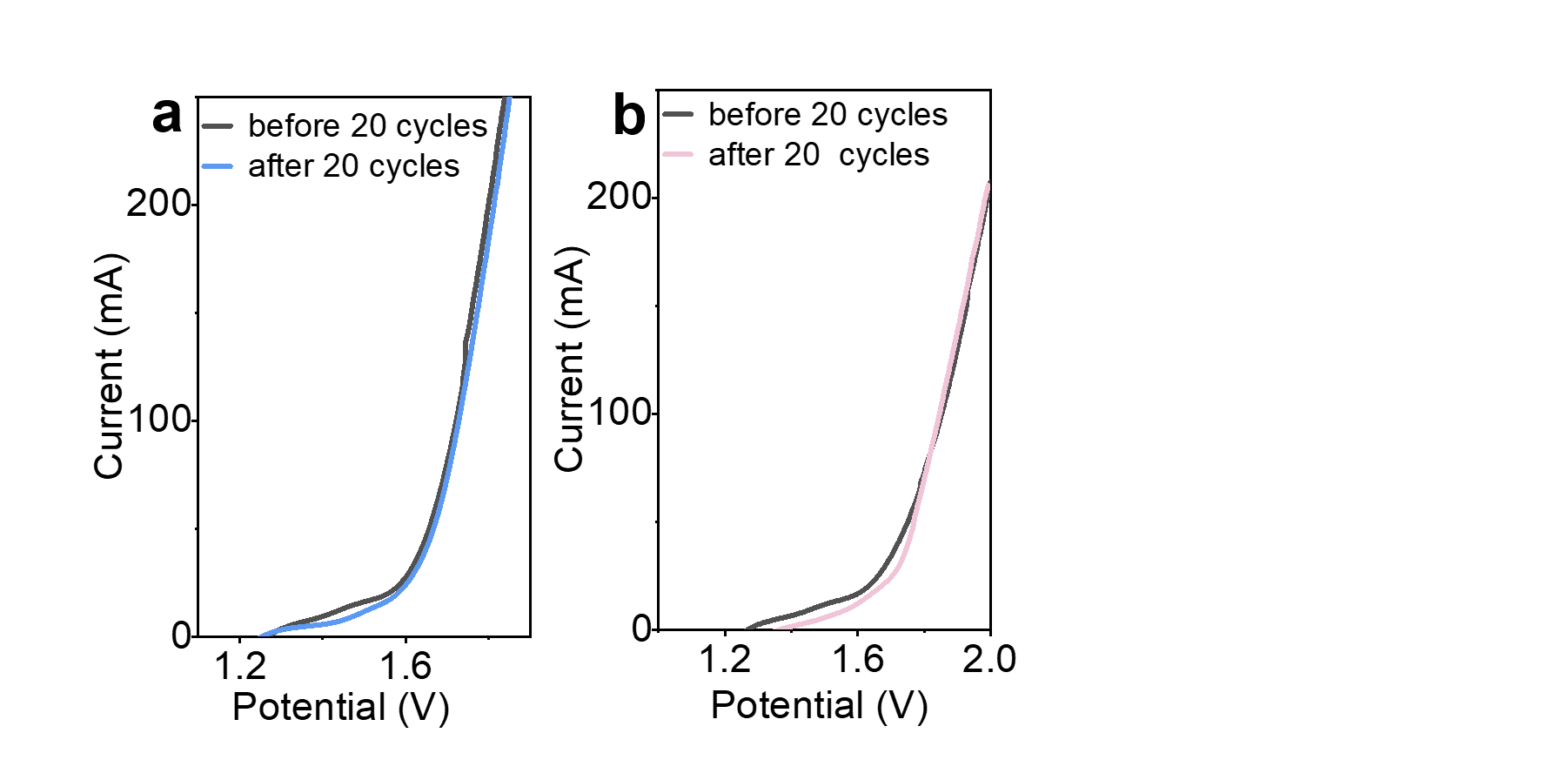


**Figure S8.** Linear sweep voltammetry (LSV) curves after electrochemical activation, with the influence of surface redox/oxide peaks eliminated. Panel (a) and (b) are OER activity of sample Mo/CoNiO_x_ and CoNiO_x_ with the reference catalyst after activation., where the curves are smooth and free from oxide peak interference, providing a more reliable evaluation of the intrinsic electrocatalytic performance.


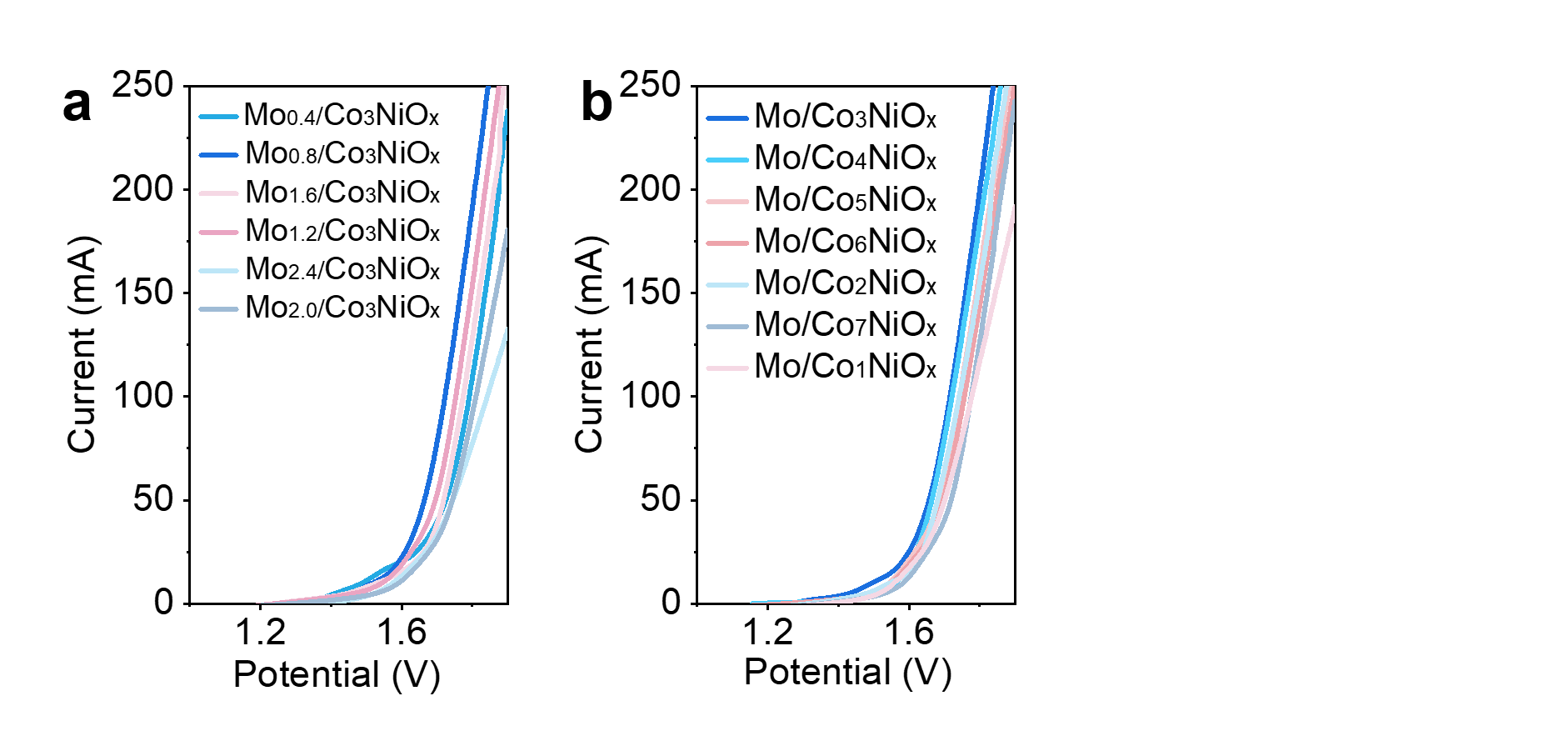


**Figure S9.** Linear sweep voltammetry (LSV) curves of catalysts with different compositions (a) the effect of varying Mo loading amounts under a fixed Co:Ni molar ratio. (b) the electrocatalytic performance at a constant Mo loading of 20% (relative to the total Co+Ni (amount of substance)) with different Co:Ni ratios (amount of substance). The results demonstrate that both Mo content and the Co/Ni ratio significantly influence the OER activity of the catalysts.


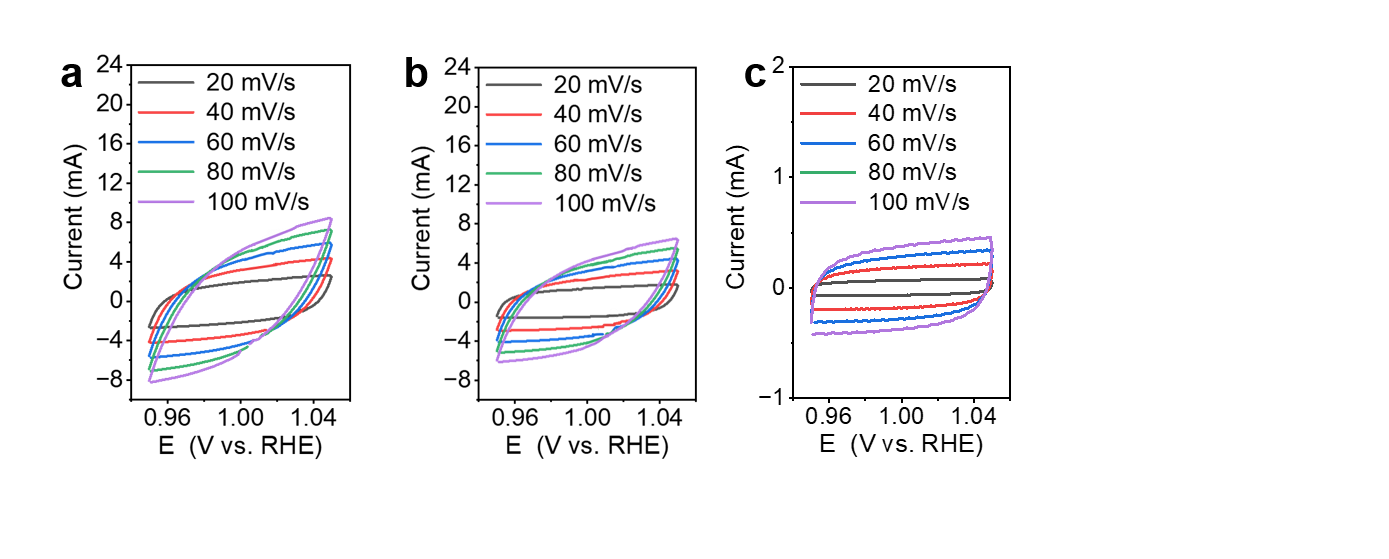


**Figure S10.** ECSA measurement of Mo/Co_3_NiO_x_ (a), Co_3_NiO_x_ (b), and RuO_2_ (c). Cyclic voltammograms were measured in a non-Faradaic region of the voltammogram at the following scan rate: 20, 40, 60, 80 and 100 mV/s. All currents were assumed to be due to capacitive charging.

**
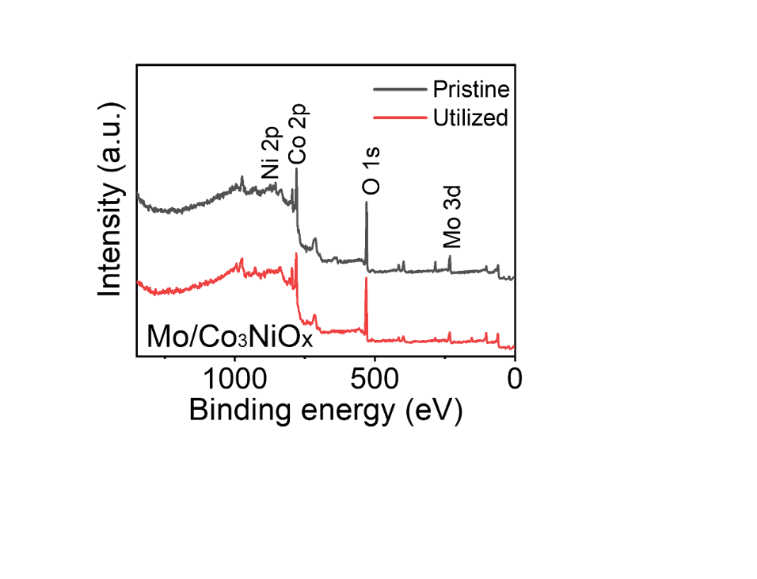
**

**Figure S11.** Full range XPS survey spectra of the pristine and utilized Mo/Co_3_NiO_x_ catalysts.

**
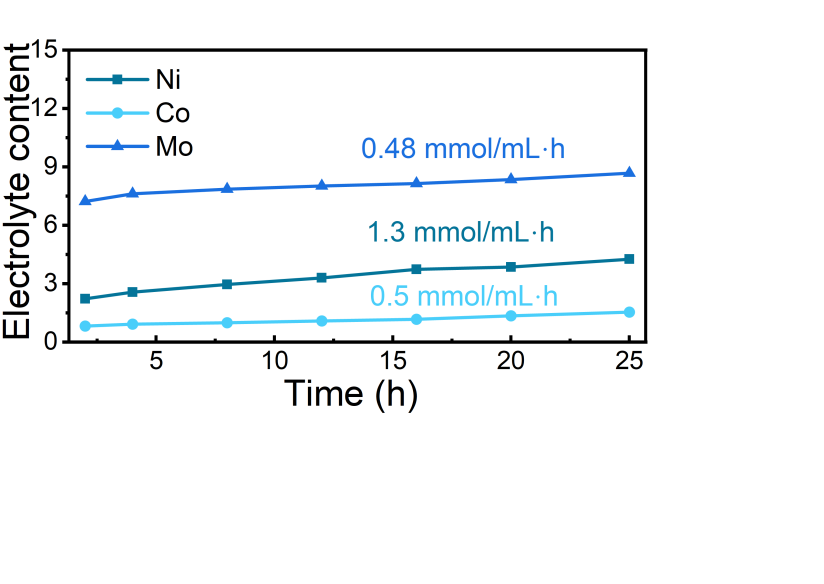
**

**Figure S12.** Time-dependent concentration variation of different elements dissolved in the electrolyte, determined by ICP analysis. The annotated values (0.48, 1.3, 0.5 mmol·mL^–1^·h^–1^) represent the average dissolution rates of the corresponding elements, highlighting their stability differences during the electrolysis process.


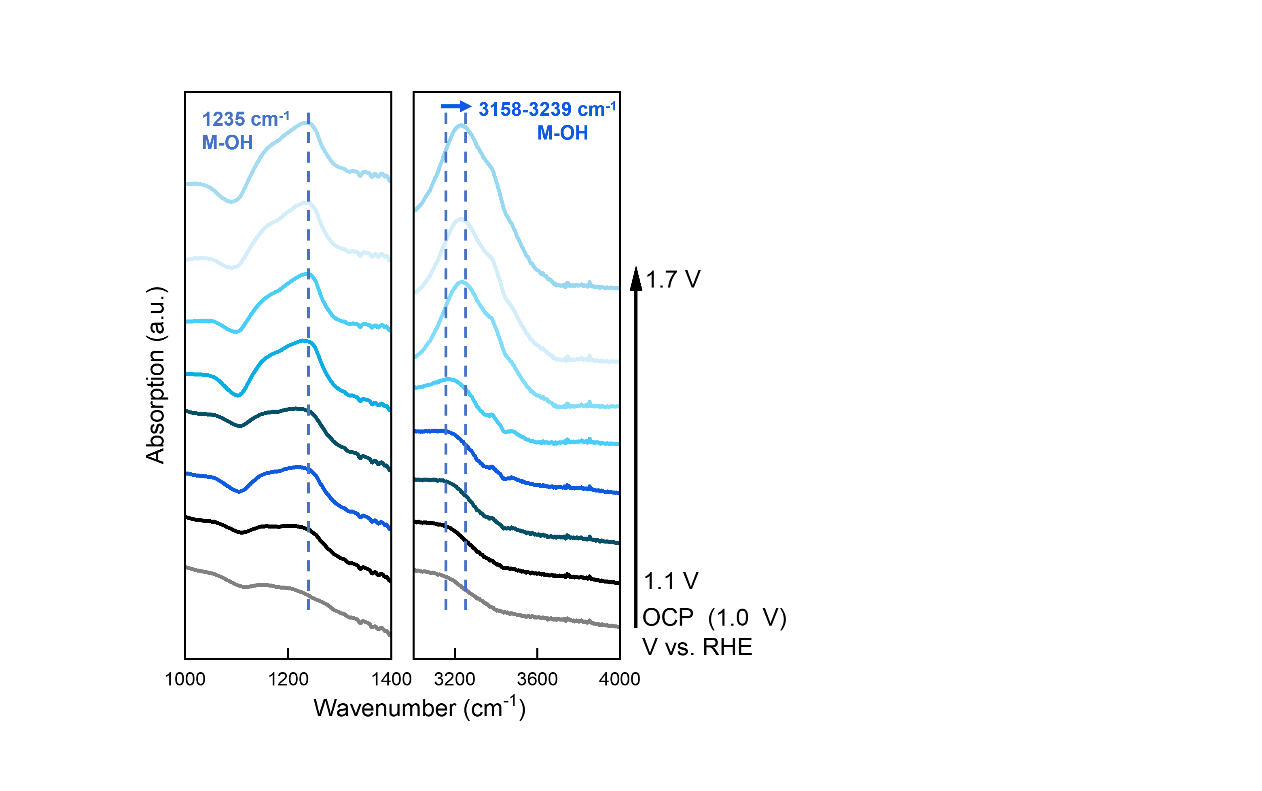


**Figure S13.** In-situ ATR-SEIRAS analysis of pristine Co_3_NiO_x_ carriers.


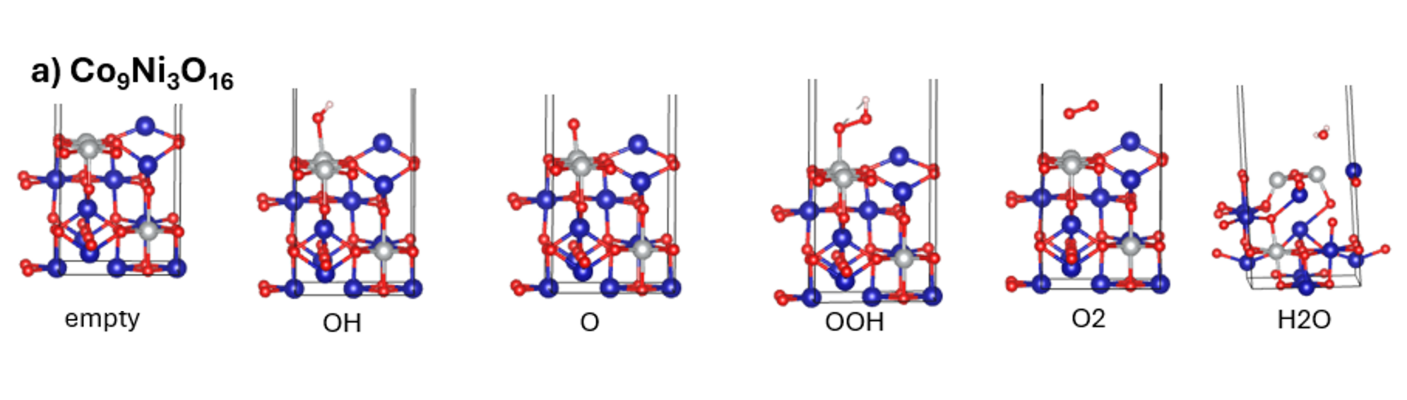


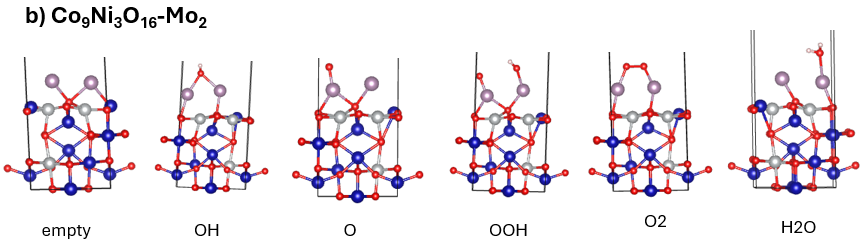


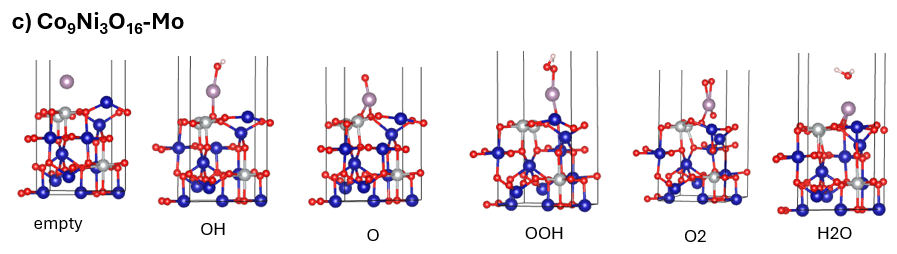


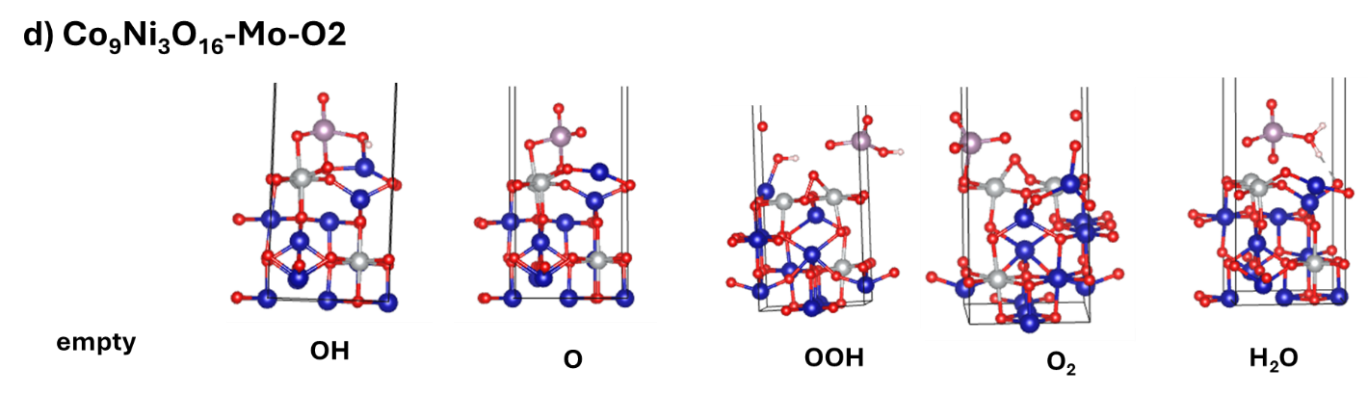


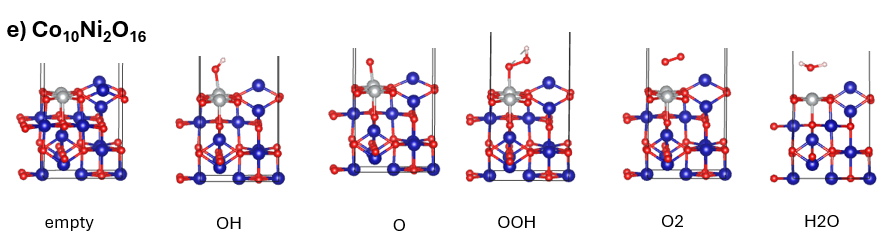


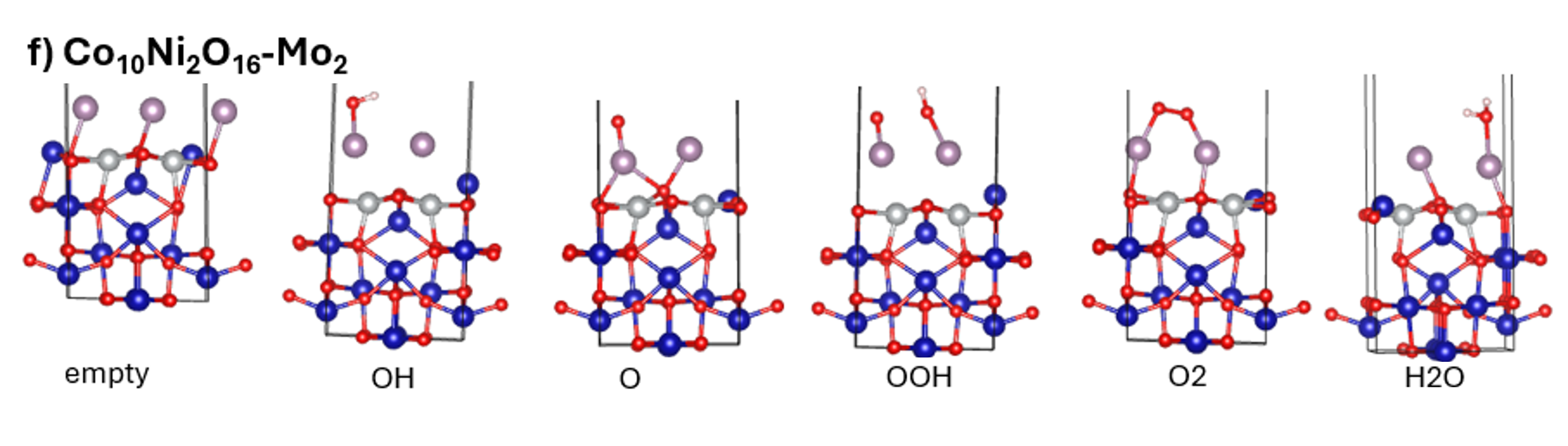


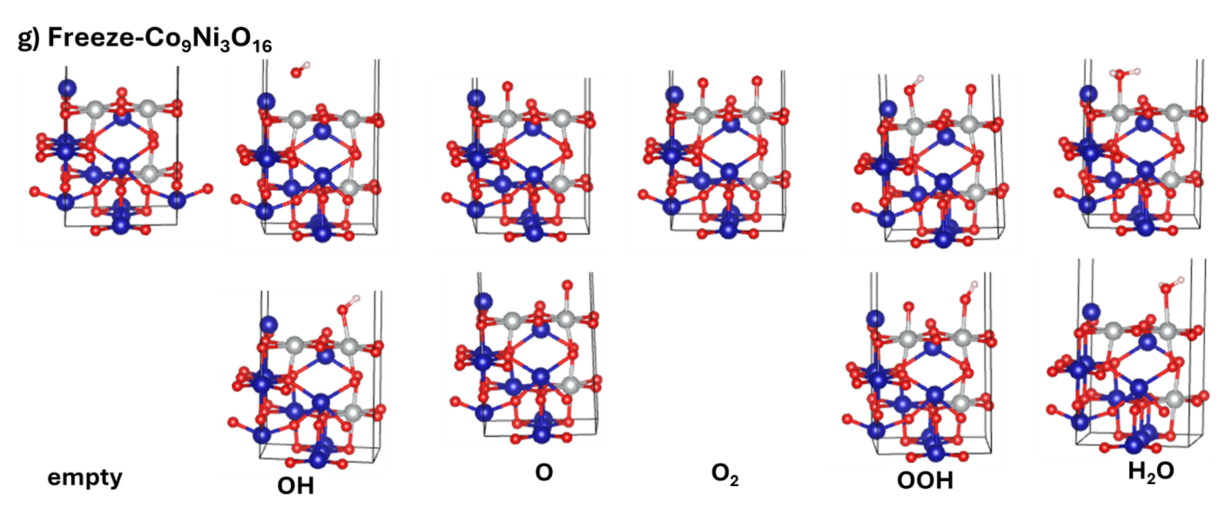


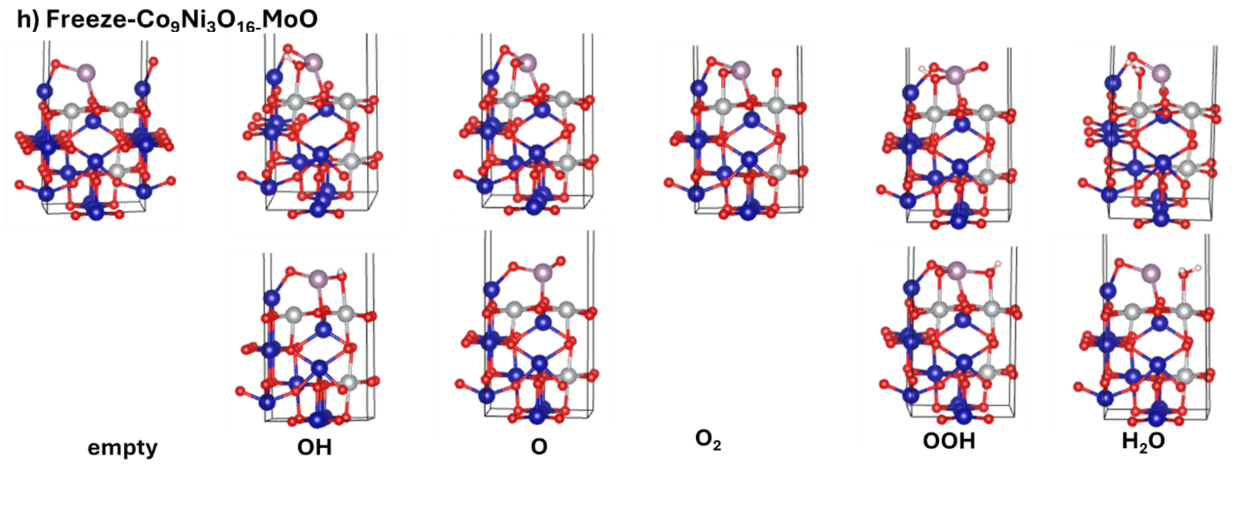


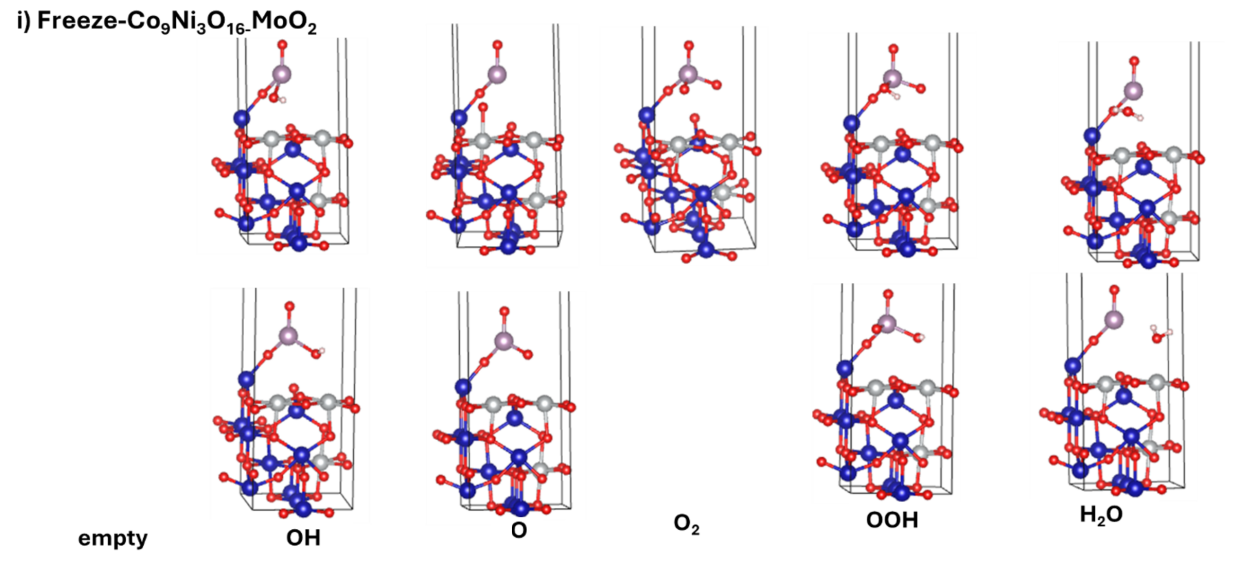


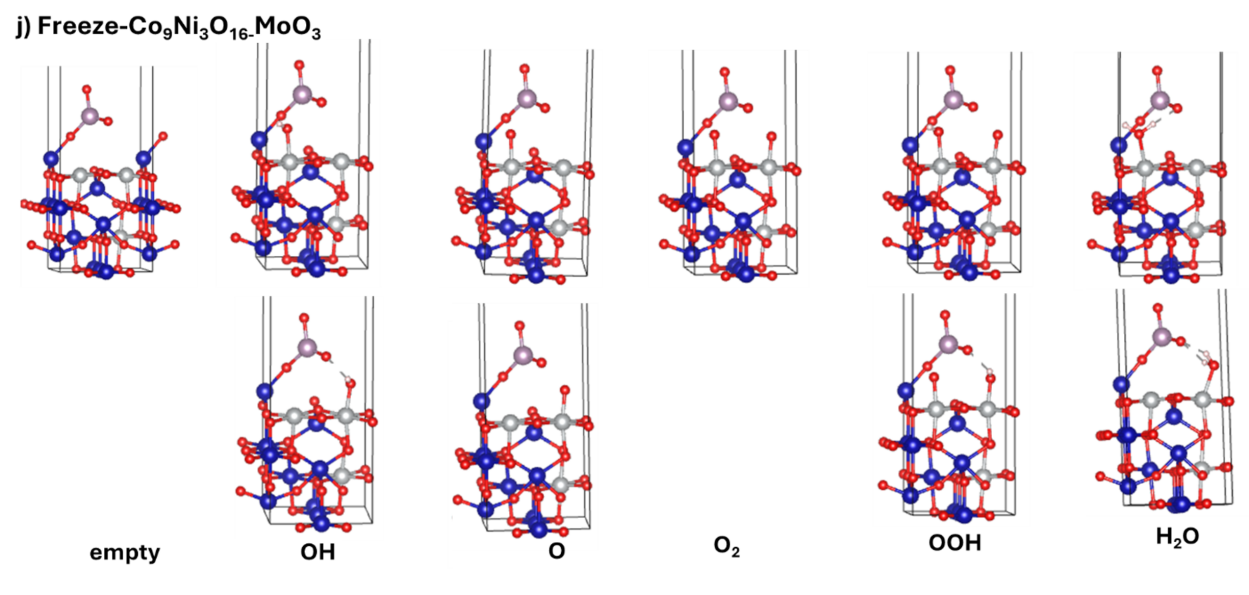


**Figure S14**. The intermediates of acidic OER on Co_3_NiO_x_ (a-d) series, Co_5_NiO_x_ series of catalysts (e-f) and MoO_n_/ Co_3_NiO_x_ series (j-k).

**
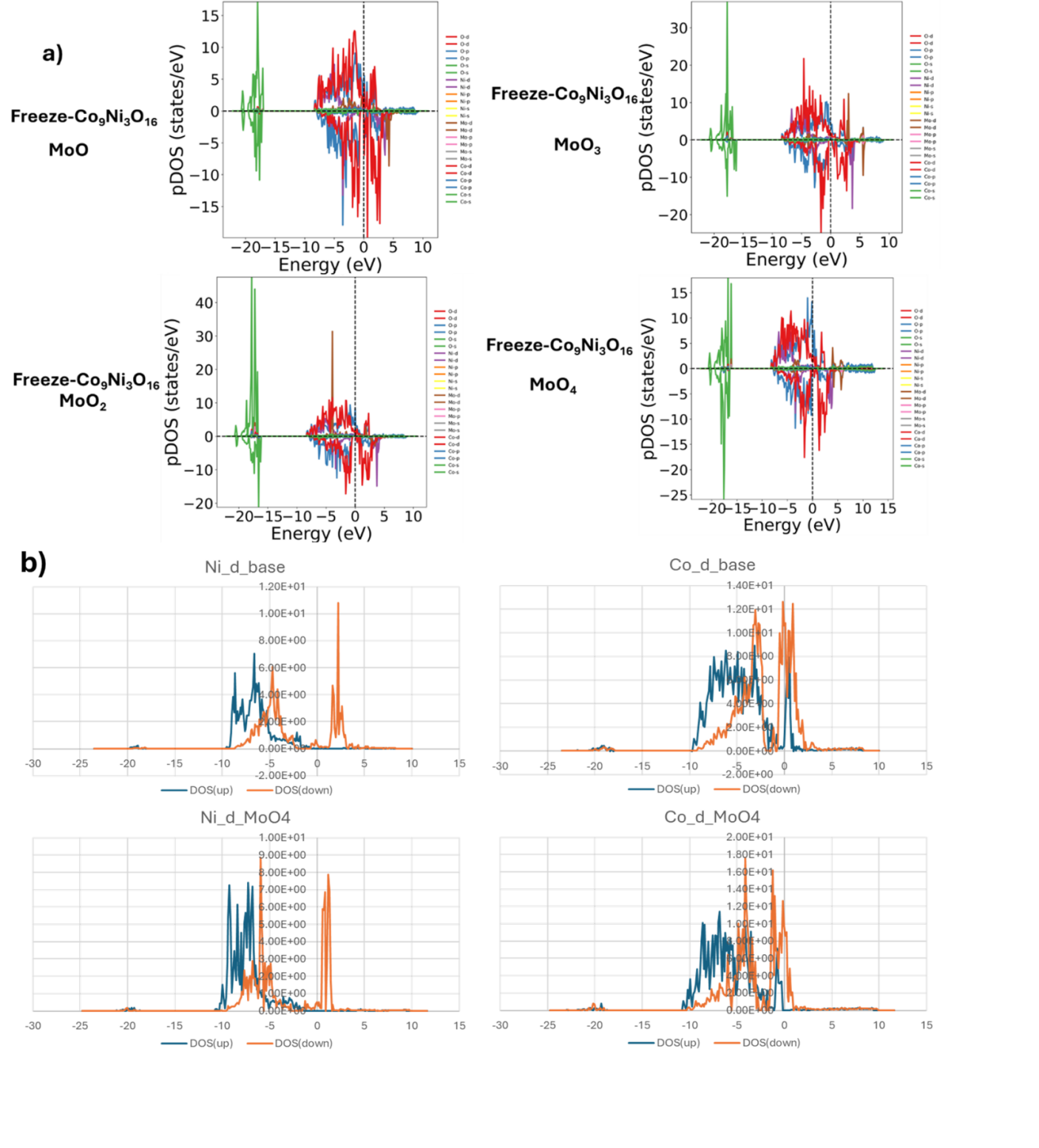
**

**
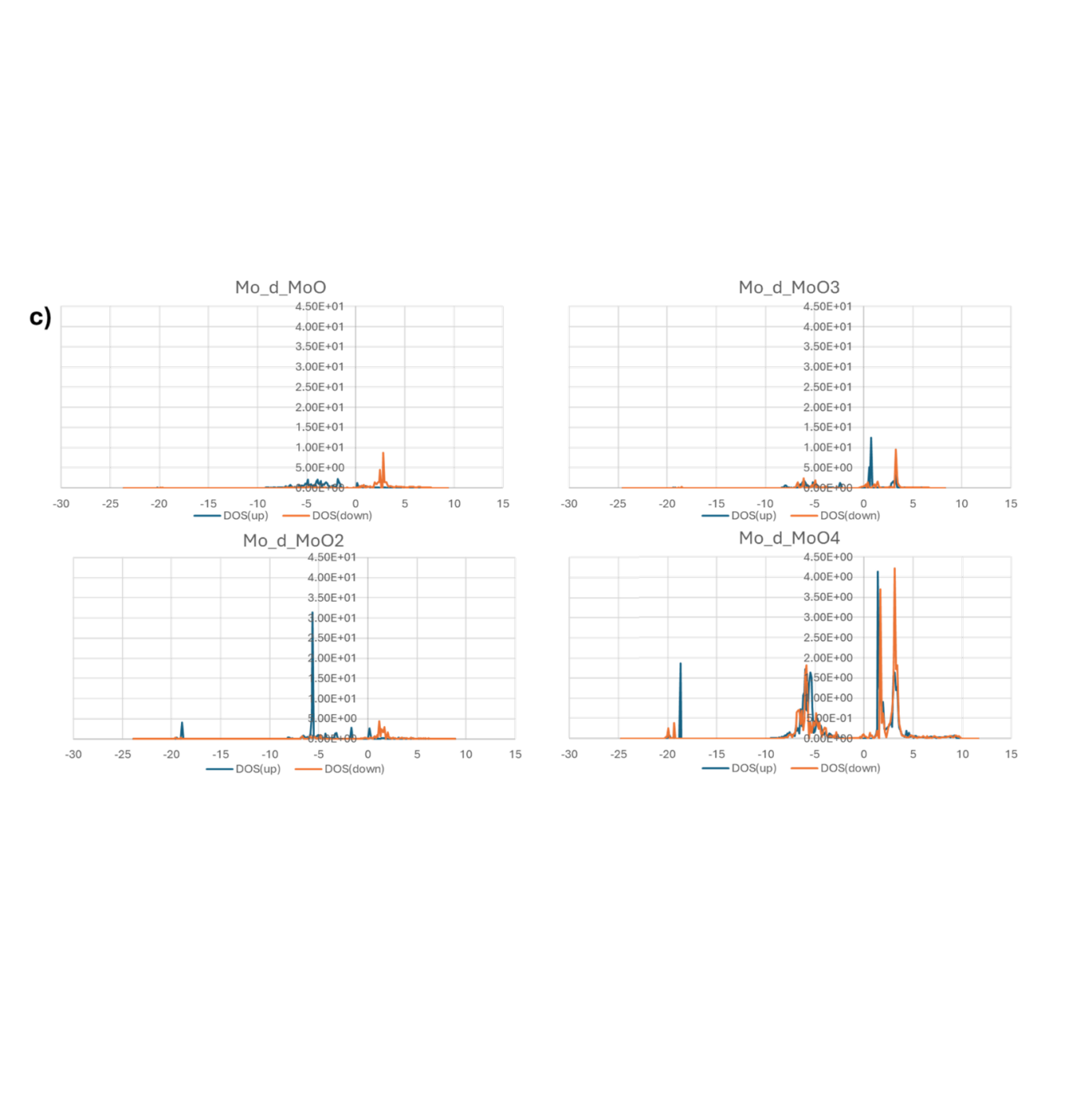
**

**Figure S15**. The projected density of states (p-DOS) plot. a) Total pDOS for MoO_n_/ Co_3_NiO_x_; b) Ni and Co d-pDOS for Co_3_NiO_x_ and MoO_4_/Co_3_NiO_x_; c) Mo d-pDOS for MoOn/Co_3_NiO_x_.

**
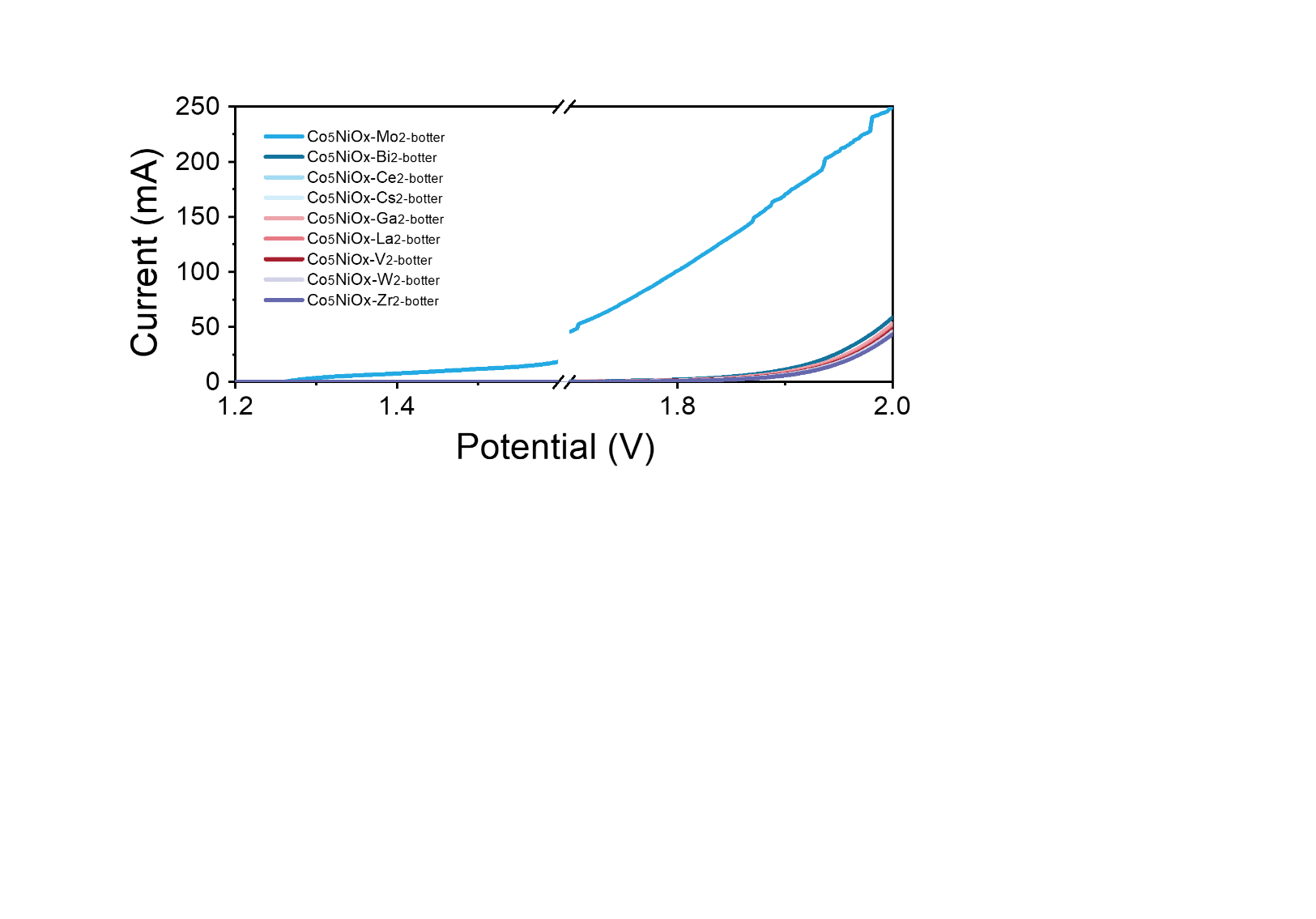
**

**Figure S16.** OER polarization curves of X_2_/Co_5_NiO_x_ with different metals (X = Mo, Ce, La, Zr, Bi, Ga, V, W).

**Table S1.** XPS analysis of Mo/Co_3_NiO_x_ and Co_3_NiO_x_.

|  | **Co^3+^%** | **Co^2+^%** | **O_L_%** | **Ov%** | **O_H_%** | **Ni^2+^%** | **Ni^3+^%** |
| --- | --- | --- | --- | --- | --- | --- | --- |
| Mo/Co_3_NiO_x_ | 49 | 51 | 48.5 | 50 | 1.5 | 72 | 28 |
| Co_3_NiO_x_ | 41 | 59 | 41 | 38 | 21 | 76.5 | 23.5 |

**Table S2.** The EIS results of catalysts.

|  | **parameter** | **Re** | **Cd** | **Rt** | **Wo** |
| --- | --- | --- | --- | --- | --- |
| Mo/Co_3_NiO_x_ | fitting data | 0.622 Ω | 2.117×10^-4^ F/cm² | 0.49 Ω | 0.1613 Ω·s^-1/2^ |
| Co_3_NiO_x_ | fitting data | 0.869 Ω | 1.475×10^-4^ F/cm² | 1.35 Ω | 6.9×10^-2^ Ω·s^-1/2^ |

Electrolyte resistance (Re): the ohmic resistance of ion conduction in the solution. It is the primary impedance of the electrolyte;

Double-layer capacitance (Cd): the double-layer capacitance at the electrode-electrolyte interface. It is caused by the distribution of ions at the electrode surface;

Charge transfer resistance (Rt): the charge transfer impedance on electrode surface. It is related to reaction kinetics: the larger value, the slower charge transfer rate and the poorer OER kinetics;

Warburg element (Wo): Warburg impedance is utilized to describe electrochemical processes controlled by diffusion. Manifesting in the low-frequency region, Warburg impedance reflects the diffusion behavior of reactants or products near the electrode surface.

**Table S3**. Summary of the OER performance in acidic electrolytes for water splitting.

| **Catalyst** | **Electrolyte** | **Performance** | | **Stability** | **Ref.** |
| --- | --- | --- | --- | --- | --- |
| Mo/Co_3_NiO_x_ | 0.5 M H_2_SO_4_ | 233 mV@10mA cm^–2^ | | 200 h@10 mA cm^–2^  >25 h@100 mA cm^–2^ | This work |
| Co_2_TiO_4_ | 0.5 M H_2_SO_4_ | 513 mV@10mA cm^–2^ | | ~5 h@~10 mA cm^–2^ | ^[2]^ |
| Co-doped 6H–SrIrO3 | 0.1 M HClO4 | 235 mV@10mA cm^–2^ | | 20 h@10 mA cm^–2^ | ^[3]^ |
| Co–RuIr | 0.1 M HClO4 | 235 mV@10mA cm^–2^ | | 25 h@10 mA cm^–2^ | ^[4]^ |
| Co0.05Fe0.95Oy | 0.3 M H_2_SO_4_ | 650 mV@10mA cm^–2^ | | 50 h@40 mA cm^–2^ | ^[5]^ |
| MnMoCoOx | 2 M H_2_SO_4_ | 305 mV@10mA cm^–2^ | | 1 h@~65 mA cm^–2^ | ^[6]^ |
| CoFePbOx | 0.5 M H_2_SO_4_ | 700 mV@10mA cm^–2^ | | 14 h@10 mA cm^–2^ | ^[7]^ |
| Co3O4/FTO | 0.5 M H_2_SO_4_ | 570 mV@10mA cm^–2^ | | 12h@10 mA cm^–2^ | ^[8]^ |
| RuO2/(Co, Mn)3O4 | 0.5 M H_2_SO_4_ | 270 mV@10mA cm^–2^ | | 24 h@10 mA cm^–2^ | ^[9]^ |
| IrCo@IrOx-nL NDs | 0.05 M H_2_SO_4_ | 247 mV@10mA cm^–2^ | | 10 h@2.5 mA cm^–2^ | ^[10]^ |
| Ni2Ta | 0.5 M H_2_SO_4_ | 570 mV@10mA cm^–2^ | | 66 h@10 mA cm^–2^ | ^[11]^ |
| NiFeP | 0.05 M H_2_SO_4_ | 540 mV@10mA cm^–2^ | 30 h@10 mA cm^–2^ | | ^[12]^ |
| 4% Er-Co_3_O_4_ | 0.5 M H_2_SO_4_ | 321 mV@10mA cm^–2^ | | 250 h@10 mA cm^–2^ | ^[13]^ |
| Ru-Co_3_O_4_ | 0.5 M H_2_SO_4_ | 250 mV@10mA cm^–2^ | | 100 h@10 mA cm^–2^ | ^[14]^ |
| Co_3_O_4-x_Fx | 0.5 M H_2_SO_4_ | 349 mV@10mA cm^–2^ | | 120 h@100 mA cm^–2^ | ^[15]^ |
| Mn1Co5Ox | 0.5 M H_2_SO_4_ | 275 mV@10mA cm^–2^ | | 260 h@100 mA cm^–2^ | ^[16]^ |
| NiMn_1.5_Co_3_O_4-δ_ | 0.5 M H2SO4 | 280 mV@10mA cm^–2^ | | 80 h@10 mA cm^–2^ | ^[17]^ |
| Co_SA_MoCeO_x_ | 0.5 M H_2_SO_4_ | 333 mV@10mA cm^–2^ | | 60 h@10 mA cm^–2^ | ^[18]^ |

**Table S4**. Comparison of the pristine and utilized Mo/Co_3_NiO_x_ by XPS.

|  | **Co^3+^%** | **Co^2+^%** | **O_L_%** | **Ov%** | **O_H_%** | **O_W_%** | **Mo^5+^%** | **Mo^6+^%** |
| --- | --- | --- | --- | --- | --- | --- | --- | --- |
| pristine | 49 | 51 | 50 | 48.5 | - | 1.5 | 81 | 19 |
| utilized | 41 | 59 | 32 | 16 | 44 | 8 | 40 | 60 |

**Table S5.** Time-dependent dissolution of elements in electrolyte (100 mA cm^–2^, ICP results).

| Conc. Units | 2 h (mg L^–1^) | 4h (mg L^–1^) | 8h (mg L^–1^) | 12h (mg L^–1^) | 16h (mg L^–1^) | 20h (mg L^–1^) | 25h (mg L^–1^) |
| --- | --- | --- | --- | --- | --- | --- | --- |
| Ni | 2.115 | 2.558 | 2.954 | 3.287 | 3.732 | 4.628 | 5.141 |
| Co | 0.817 | 0.913 | 0.986 | 1.078 | 1.163 | 1.344 | 1.536 |
| Mo | 6.817 | 7.613 | 7.850 | 8.016 | 8.135 | 8.345 | 8.671 |

**Table S6.** Elemental changes pre- and post-stability test (100 mA cm^-2^, ICP).

| Conc. Units | Pre (mg L^–1^) | Post (mg L^–1^) |
| --- | --- | --- |
| Co | 28.33 | 21.55 |
| Ni | 13.54 | 5.23 |
| Mo | 18.45 | 13.11 |

ICP results showing the elemental concentrations of Co, Ni, and Mo in Mo/Co_3_NiO_x_ catalysts before and after the stability test at 100 mA cm^–2^. A pronounced decrease in Ni content (61% loss) is observed, in contrast to the moderate decreases in Co (24%) and Mo (29%), indicating that Ni leaching is the dominant degradation pathway under acidic OER conditions

**Table S7.** The one-electron transfer Gibbs free energies and binding energy for intermediates on Co_3_NiO_x_ series of catalysts.

| ΔG (298.15 K) (eV) | **η_OER_ (V)** | **OH*** | **O*** | **OOH*** | **O_2_*** |
| --- | --- | --- | --- | --- | --- |
| Co_3_NiO_x_ | **4.95** | 4.95 | 3.75 | -11.57 | -0.89 |
| MoO_2_/Co_3_NiO_x_ | **3.62** | 2.87 | 3.19 | -9.23 | -3.84 |
| Mo/Co_3_NiO_x_ | **2.68** | 2.68 | 0.77 | -5.63 | 2.05 |
| Mo_2_/Co_3_NiO_x_ | **5.19** | 2.20 | 0.20 | -11.54 | -4.89 |

| Binding energy (eV) | **η_OER_ (V)** | **OH*** | **O*** | **OOH*** | **O*-OH*** | **3.2-(O*-OH*)** |
| --- | --- | --- | --- | --- | --- | --- |
| Co_3_NiO_x_ | **2.70** | 4.94 | 8.87 | 11.18 | 3.93 | -0.73 |
| MoO_2_/Co_3_NiO_x_ | **2.14** | 0.84 | 4.22 | 8.87 | 3.37 | -0.17 |
| Mo/Co_3_NiO_x_ | **1.02** | -0.48 | 0.47 | 8.73 | 0.95 | 2.25 |
| Mo_2_/Co_3_NiO_x_ | **1.59** | 1.52 | 1.90 | 4.25 | 0.38 | 2.82 |

**Table S8.** The one-electron transfer Gibbs free energies and binding energy for intermediates on MoO_n_/Co_3_NiO_x_ series of catalysts.

| ΔG(298.15 K) (eV) | **η_OER_ (V)** | **OH*** | **O*** | **OOH*** | **O_2_*** |
| --- | --- | --- | --- | --- | --- |
| Co_3_NiO_x_ | **4.67** | 4.67 | 2.43 | 3.82 | 4.57 |
| Mo/Co_3_NiO_x_ | **2.69** | 1.04 | 2.49 | 0.76 | 2.69 |
| MoO_2_/Co_3_NiO_x_ | **4.61** | 2.54 | 4.61 | -1.86 | 4.08 |
| MoO_3_/Co_3_NiO_x_ | **4.81** | 4.30 | 3.91 | 3.43 | 4.81 |
| MoO_4_/Co_3_NiO_x_ | **4.20** | 4.20 | 3.10 | 3.73 | 3.83 |
| alternative position |  |  |  |  |  |
| Co_3_NiO_x_ | **4.58** | 4.58 | 3.18 | 4.36 | 4.13 |
| Mo/Co_3_NiO_x_ | **4.64** | 2.58 | 1.40 | 4.64 | -0.71 |
| MoO_2_/Co_3_NiO_x_ | **3.73** | 1.40 | 3.00 | 0.53 | 3.73 |
| MoO_3_/Co_3_NiO_x_ | **4.86** | 4.34 | 4.44 | 4.86 | 3.31 |
| MoO_4_/Co_3_NiO_x_ | **4.36** | 4.20 | 3.16 | 4.36 | 2.58 |

| Binding energy (eV) | **η_OER_ (V)** | **OH*** | **O*** | **OOH*** | **O*-OH*** | **3.2-(O*-OH*)** |
| --- | --- | --- | --- | --- | --- | --- |
| Co_3_NiO_x_ | **1.38** | 6.98 | 9.59 | 13.59 | 2.61 | 0.59 |
| Mo/Co_3_NiO_x_ | **1.44** | 0.86 | 3.53 | 4.48 | 2.67 | 0.53 |
| MoO_2_/Co_3_NiO_x_ | **3.56** | 2.62 | 7.41 | 5.74 | 4.79 | -1.59 |
| MoO_3_/Co_3_NiO_x_ | **2.86** | 4.66 | 8.76 | 12.38 | 4.09 | -0.89 |
| MoO_4_/Co_3_NiO_x_ | **2.05** | 4.82 | 8.10 | 12.02 | 3.28 | -0.08 |
| alternative position |  |  |  |  |  |  |
| Co_3_NiO_x_ | **2.13** | 6.13 | 9.49 | 14.04 | 3.36 | -0.16 |
| Mo/Co_3_NiO_x_ | **0.39** | 1.47 | 3.05 | 7.87 | 1.58 | 1.62 |
| MoO_2_/Co_3_NiO_x_ | **1.96** | 2.19 | 5.38 | 6.10 | 3.19 | 0.01 |
| MoO_3_/Co_3_NiO_x_ | **3.39** | 4.21 | 8.83 | 13.88 | 4.62 | -1.42 |
| MoO_4_/Co_3_NiO_x_ | **2.11** | 5.38 | 8.72 | 13.27 | 3.34 | -0.14 |

**Table S9.** The integrated electron density on Mo site for MoO_n_/Co_3_NiO_x_ series of catalysts.

| **Integrated density** | **s** | **p** | **d** | **tot** |
| --- | --- | --- | --- | --- |
| **MoO/Co_3_NiO_x_** |  |  |  |  |
| total charge | 0.367 | 0.245 | 4.228 | 4.840 |
| magnetization | 0.173 | 0.019 | 3.407 | 3.599 |
| **MoO_2_/Co_3_NiO_x_** |  |  |  |  |
| total charge | 0.409 | 0.468 | 4.396 | 5.273 |
| magnetization | 0.159 | 0.025 | 2.287 | 2.471 |
| **MoO_3_/Co_3_NiO_x_** |  |  |  |  |
| total charge | 0.545 | 0.717 | 4.461 | 5.723 |
| magnetization | 0.144 | 0.014 | 0.864 | 1.022 |
| **MoO_4_/Co_3_NiO_x_** |  |  |  |  |
| total charge | 0.623 | 0.854 | 4.623 | 6.100 |
| magnetization | 0.006 | 0.006 | 0.147 | 0.159 |

**Table S10.** The Gibbs free energies (ΔG, eV) and binding energy for intermediates on X_2_/Co_5_NiO_x_ series of catalysts.

| ΔG (298.15 K) (eV) | **η_OER_ (V)** |  | **OH*** | **O*** | **OOH*** | **O_2_*** |
| --- | --- | --- | --- | --- | --- | --- |
| Co_10_Ni_2_O_16_ | **4.25** |  | 2.64 | 4.25 | -12.79 | 2.83 |
| Mo_2_/Co_10_Ni_2_O_16_ | **4.15** |  | 4.15 | -1.09 | -6.85 | -0.90 |
| Ce_2_/Co_10_Ni_2_O_16_ | **2.03** |  | 0.19 | 0.40 | -13.62 | 2.03 |
| La_2_/Co_10_Ni_2_O_16_ | **0.03** |  | -1.61 | 0.03 | -9.23 | -0.91 |
| Zr_2_/Co_10_Ni_2_O_16_ | **3.29** |  | -0.71 | 3.29 | -12.92 | -2.48 |
| Bi_2_/Co_10_Ni_2_O_16_ | **3.46** |  | 3.46 | 0.73 | -7.07 | 0.97 |
| Ga_2_/Co_10_Ni_2_O_16_ | **3.77** |  | 3.77 | 1.30 | -7.79 | 1.45 |
| V_2_/Co_10_Ni_2_O_16_ | **5.88** |  | 5.88 | -3.99 | -12.29 | 5.58 |
| W_2_/Co_10_Ni_2_O_16_ | **4.59** |  | 1.25 | 3.26 | -16.32 | 4.59 |

| Binding energy (eV) | **η_OER_ V)** | **OH*** | **O*** | **OOH*** | **O*-OH*** | **3.2-(O*-OH*)** |
| --- | --- | --- | --- | --- | --- | --- |
| Co_10_Ni_2_O_16_ | **4.43** | 3.70 | 8.13 | 9.23 | 4.43 | -1.23 |
| Mo_2_/Co_10_Ni_2_O_16_ | **4.11** | 4.41 | 3.50 | 10.53 | -0.91 | 4.11 |
| Ce_2_/Co_10_Ni_2_O_16_ | **2.62** | 2.69 | 3.27 | 3.53 | 0.58 | 2.62 |
| La_2_/Co_10_Ni_2_O_16_ | **2.99** | -2.17 | -1.96 | 2.69 | 0.21 | 2.99 |
| Zr_2_/Co_10_Ni_2_O_16_ | **3.47** | 0.82 | 4.29 | 5.26 | 3.47 | -0.27 |
| Bi_2_/Co_10_Ni_2_O_16_ | **2.29** | 2.91 | 3.82 | 10.63 | 0.91 | 2.29 |
| Ga_2_/Co_10_Ni_2_O_16_ | **1.71** | 3.49 | 4.98 | 11.08 | 1.49 | 1.71 |
| V_2_/Co_10_Ni_2_O_16_ | **7.01** | 6.70 | 2.89 | 4.48 | -3.81 | 7.01 |
| W_2_/Co_10_Ni_2_O_16_ | **3.45** | 0.33 | 3.78 | 1.34 | 3.45 | -0.25 |

**Reference**

[1] I. C. Man, H.-Y. Su, F. Calle-Vallejo, H. A. Hansen, J. I. Martínez, N. G. Inoglu, J. Kitchin, T. F. Jaramillo, J. K. Nørskov, J. Rossmeisl, *ChemCatChem* **2011**, *3*, 1159-1165.

[2] S. Anantharaj, K. Karthick, S. Kundu, *Inorganic Chemistry* **2019**, *58*, 8570-8576.

[3] J.-W. Zhao, K. Yue, H. Zhang, S.-Y. Wei, J. Zhu, D. Wang, J. Chen, V. Y. Fominski, G.-R. Li, *Nat. Commun.* **2024**, *15*, 2928.

[4] J. Shan, T. Ling, K. Davey, Y. Zheng, S. Z. Qiao, *Adv. Mater.* **2019**, *31*, e1900510.

[5] W. L. Kwong, C. C. Lee, A. Shchukarev, J. Messinger, *Chem. Commun. (Cambridge, U. K.)* **2019**, *55*, 5017-5020.

[6] D. Delgado, M. Minakshi, J. McGinnity, D. J. Kim, *Sci Rep* **2015**, *5*, 15208.

[7] M. Chatti, J. L. Gardiner, M. Fournier, B. Johannessen, T. Williams, T. R. Gengenbach, N. Pai, C. Nguyen, D. R. MacFarlane, R. K. Hocking, A. N. Simonov, *Nat. Catal.* **2019**, *2*, 457-465.

[8] J. S. Mondschein, J. F. Callejas, C. G. Read, J. Y. C. Chen, C. F. Holder, C. K. Badding, R. E. Schaak, *Chem. Mater.* **2017**, *29*, 950-957.

[9] C. Zhao, Y. Wang, Z. Li, W. Chen, Q. Xu, D. He, D. Xi, Q. Zhang, T. Yuan, Y. Qu, J. Yang, F. Zhou, Z. Yang, X. Wang, J. Wang, J. Luo, Y. Li, H. Duan, Y. Wu, Y. Li, *Joule* **2019**, *3*, 584-594.

[10] G. Meng, W. Sun, A. A. Mon, X. Wu, L. Xia, A. Han, Y. Wang, Z. Zhuang, J. Liu, D. Wang, Y. Li, *Adv. Mater.* **2019**, *31*, e1903616.

[11] J. S. Mondschein, K. Kumar, C. F. Holder, K. Seth, H. Kim, R. E. Schaak, *Inorg. Chem.* **2018**, *57*, 6010-6015.

[12] L. Hu, X. Zeng, X. Wei, H. Wang, Y. Wu, W. Gu, L. Shi, C. Zhu, *Appl. Catal. B* **2020**, *273*, 119014.

[13] S. Pan, H. Li, T. Wang, Y. Fu, S. Wang, Z. Xie, L. Wei, H. Li, N. Li, *ACS Catal.* **2024**, *14*, 13814-13824.

[14] Y. Yang, Y. Xu, H. Liu, Q. Zhang, B. Liu, M. Yang, H. Dai, Z. Ke, D. He, X. Feng, X. Xiao, *Nano Res.* **2024**, *17*, 5922-5929.

[15] Y. Wang, P. Guo, J. Zhou, B. Bai, Y. Li, M. Li, P. Das, X. Wu, L. Zhang, Y. Cui, J. Xiao, Z.-S. Wu, *Energy Environ. Sci.* **2024**, *17*, 8820-8828.

[16] J. Zhang, A. Raza, Y. Zhao, S. Guo, Z. U. D. Babar, L. Xu, C. Cao, G. Li, *J. Mater. Chem. A* **2023**, *11*, 25345-25355.

[17] H. Zhao, L. Zhu, J. Yin, J. Jin, X. Du, L. Tan, Y. Peng, P. Xi, C. H. Yan, *Angew. Chem. Int. Ed. Engl.* **2024**, *63*, e202402171.

[18] J. Liu, T. Wang, Z. Lin, M. Liao, S. Liu, S. Wang, Z. Cai, H. Sun, Y. Shen, Y. Huang, Q. Li, *Energy Environ. Sci.* **2024**, *17*, 3088-3098.
